# The compensatory effect of education as revealed by resting-state electroencephalographic alpha rhythms in patients with dementia due to Parkinson’s disease: findings from an exploratory study

**DOI:** 10.1007/s11357-025-01703-9

**Published:** 2025-06-11

**Authors:** Susanna Lopez, Claudio Del Percio, Roberta Lizio, Giuseppe Noce, Dharmendra Jakhar, Andrea Soricelli, Marco Salvatore, Bahar Güntekin, Görsev Yener, Federico Massa, Dario Arnaldi, Francesco Famà, Matteo Pardini, Raffaele Ferri, Filippo Carducci, Bartolo Lanuzza, Fabrizio Stocchi, Laura Vacca, Chiara Coletti, Moira Marizzoni, John-Paul Taylor, Lutfu Hanoğlu, Nesrin Helvacı Yılmaz, İlayda Kıyı, Yağmur Özbek-İşbitiren, Anita D’Anselmo, Laura Bonanni, Roberta Biundo, Fabrizia D’Antonio, Giuseppe Bruno, Angelo Antonini, Franco Giubilei, Sofia Cuoco, Paolo Barone, Giovanni B. Frisoni, Rossella Rotondo, Francesca De Pandis, Claudio Babiloni

**Affiliations:** 1 Department of Physiology and Pharmacology “Vittorio Erspamer”, Sapienza University of Rome, P. Le A. Moro 5, 00185 Rome, Italy; 2 IRCCS Synlab SDN, Naples, Italy; 3 Department of Medical, Movement and Well-Being Sciences, University of Naples Parthenope, Naples, Italy; 4 Department of Biophysics, School of Medicine, Istanbul Medipol University, Istanbul, Turkey; 5 Faculty of Medicine, Izmir University of Economics, Izmir, Turkey; 6 Dipartimento Di Neuroscienze, Oftalmologia, Genetica, Riabilitazione e Scienze Materno-Infantili (DiNOGMI), Università Di Genova, Genoa, Italy; 7 Present Address: Clinica Neurologica, IRCCS Ospedale Policlinico San Martino, Genoa, Italy; 8 Neurofisiopatologia, IRCCS Ospedale Policlinico San Martino, Genoa, Italy; 9 Oasi Research Institute – IRCCS, Troina, Italy; 10 IRCCS San Raffaele, Rome, Italy; 11 Telematic University San Raffaele Rome, Rome, Italy; 12 Biological Psychiatry Unit, IRCCS Istituto Centro San Giovanni Di Dio Fatebenefratelli, Brescia, Italy; 13 Translational and Clinical Research Institute, Faculty of Medical Sciences, Newcastle University, Newcastle upon Tyne, UK; 14 Department of Neurology, School of Medicine, Istanbul Medipol University, Istanbul, Turkey; 15 Parkinson’s Disease and Movement Disorders Center (PARMER), Istanbul Medipol University, Istanbul, Turkey; 16 Health Sciences Institute, Department of Neurosciences, Dokuz Eylül University, Izmir, Turkey; 17 Department of Aging Medicine and Sciences, University “G. d’Annunzio” of Chieti-Pescara, Chieti, Italy; 18 Parkinson and Movement Disorders Unit, Study Center for Neurodegeneration (CESNE), Center for Rare Neurological Diseases (ERN RND), Department of Neuroscience, University of Padua, Padua, Italy; 19 Department of General Psychology, University of Padova, Padua, Italy; 20 Department of Human Neurosciences, Sapienza University of Rome, Rome, Italy; 21 Department of Neuroscience, Mental Health, and Sensory Organs, Sapienza University of Rome, Rome, Italy; 22 Department of Medicine, Surgery and Dentistry “Scuola Medica Salernitana”, Neuroscience Section, University of Salerno, Baronissi, Italy; 23 Laboratory of Neuroimaging of Aging (LANVIE), University of Geneva, Geneva, Switzerland; 24 Geneva Memory Center, Department of Rehabilitation and Geriatrics, Geneva University Hospitals, Geneva, Switzerland; 25 Hospital San Raffaele Cassino, Cassino, FR Italy

**Keywords:** Parkinson’s disease dementia (PDD), Lewy body dementia (DLB), Alpha resting-state electroencephalographic (EEG) rhythms, Exact low-resolution brain electromagnetic source tomography (eLORETA), Education, Cognitive reserve

## Abstract

Here, we investigated whether educational attainment influences the neurophysiological mechanisms underlying vigilance regulation, as reflected in resting-state eyes-closed electroencephalographic (rsEEG) rhythms, in patients with dementia due to Parkinson’s (PDD) and Lewy body disease (DLB). Clinical, demographic, and rsEEG data were obtained from an international database, including PDD patients (*N* = 75), DLB patients (*N* = 50), and cognitively unimpaired older controls (Healthy; *N* = 54). Each group was partitioned into low (Edu-) and high (Edu+) educational attainment subgroups, matched for age, sex, and cognitive-motor status. We analyzed rsEEG rhythms across the individual delta, theta, and alpha frequency bands. Cortical rsEEG source topography was estimated using eLORETA freeware. In the Healthy group, Edu+ participants exhibited significantly greater widespread rsEEG alpha source activities compared to Edu- participants, possibly reflecting neuroprotective neurophysiological mechanisms. Conversely, in the PDD group, Edu+ patients showed lower widespread rsEEG alpha source activities than Edu- patients, possibly indicating compensatory mechanisms. No significant differences in rsEEG source activities were observed between DLB-Edu+ and DLB-Edu- patients. Educational attainment may be associated with compensatory mechanisms that counteract the abnormal neurophysiological processes underlying rsEEG alpha rhythms and vigilance regulation in PDD patients, but not in DLB patients. Future studies combining rsEEG and neuroimaging techniques should investigate the metabolic and functional connectivity correlates of these putative compensatory mechanisms in the PDD brain. Early education may be a key investment for national governments, especially in low-income countries, to prevent the cognitive deficits of Parkinson’s disease along aging, thereby reducing the unbearable social and economic burden.

**Supplementary Information:**

The online version contains supplementary material available at 10.1007/s11357-025-01703-9.

## Introduction

According to the World Health Organization facts (https://www.who.int/), five million people suffer from senile dementia, with about 60% of them due to Alzheimer’s disease (AD), which is caused by brain amyloidosis and tauopathy. Approximately 20% of dementia cases are attributed to Parkinson’s disease dementia (PDD) and dementia with Lewy bodies (DLB). These neurodegenerative diseases are characterized by intraneural inclusions of Lewy bodies (primarily composed of α-synuclein protein) and neurites in the basal ganglia (especially in PDD) and cortical regions (especially in DLB) [1, 2], leading to axonal dysfunction, neuronal loss, and brain neurodegeneration. Both diseases are marked by dopamine depletion in basal ganglia circuits and cholinergic depletion from forebrain cholinergic neurons [3–5].

In Parkinson’s disease (PD), motor symptoms such as resting tremor, rigidity, or bradykinesia are early core clinical manifestations, typically preceding the gradual onset of cognitive deficits, which may eventually lead to dementia [1]. In contrast, DLB patients often present cognitive deficits before or concurrently with motor symptoms [1]. Furthermore, DLB patients are more likely to experience visual hallucinations, marked diurnal cognitive fluctuations, and rapid-eye-movement sleep behavior disorder (RBD) [6]. In both PDD and DLB, cognitive deficits primarily affect executive, attentional, and verbal functions [1, 7–10].

The individual variability of neurodegenerative disease’s course from the onset of clinical manifestations to dementia depends on various factors, including the neurobiological and neurophysiological features preventing or contrasting the disease, which form a sort of brain cognitive reserve (CR). Such a reserve may be developed over a person’s lifetime in relation to high educational attainment and the related intellectual activities in work occupation, cultural interests, and creative/leisure experiences [11]. The physiological bases of CR remain unclear. CR may be associated with enhanced neurogenesis, synaptic plasticity, neuronal efficiency in neuromodulation systems, and the brain’s ability to reorganize functional connectivity flexibly to compensate for neuropathological and neurodegenerative processes [11–16]. CR cannot be measured directly but can be estimated using proxy factors such as general intelligence, education level, and the intellectual demands of one’s occupation over a lifetime, often combined through comprehensive tests and questionnaires [15, 16].

A full and longitudinal characterization of all these factors is essential to better interpret their synergic contributions to CR. Educational attainment is frequently used as a simple and effective CR proxy in scientific research due to its strong association with various factors that enhance brain resilience. Education has a clear and significant impact on brain health, making it an essential early-life strategy for preventing neurodegenerative disorders [17]. Formal educational attainment is an important indicator of socioeconomic disparities [18–20] significantly influencing brain health [20–22], providing a robust reserve against cognitive decline, and reducing the risk of developing dementia [23]. Extensive evidence shows that enhanced educational attainment effectively counters the adverse effects of brain damage in conditions such as Alzheimer’s disease (AD), frontotemporal dementia (FTD), dementia with Lewy bodies (DLB), and other neurological and psychiatric disorders [17].

CR was initially introduced to explain better-than-expected cognitive performance in Alzheimer’s disease dementia (ADD) patients despite significant brain neuropathology observed through autoptic histological analyses [24] and in vivo biomarkers of AD neuropathology [11, 15, 25]. Specifically, CR’s *neuroprotective mechanisms* have been hypothesized to explain why older adults with higher CR (Edu+) experience a delayed onset of cognitive deficits compared to those with lower CR (Edu-); at the same time, CR’s *compensatory (resilient) mechanisms* have been hypothesized to explain why Edu+ patients maintain cognitive function despite greater brain atrophy and hypometabolism, as shown by MRI and PET biomarkers [11, 15, 26–37]. The compensatory mechanism due to education attainment could also explain the results on clinical-cognitive status and autoptic neuropathological burden (e.g., neocortical and hippocampal neuritic plaques, neocortical cerebral amyloid angiopathy, and Braak stage for tau and tangles) obtained in hundreds of older patients with dementia by the EClipSE Collaborative Members [38].

CR’s effects are not limited to cognitive function and brain integrity, as revealed by neuroimaging techniques. They also influence the brain’s neurophysiological oscillatory mechanisms that regulate cortical arousal and vigilance during wakefulness, which are reflected by changes in electroencephalographic (rsEEG) rhythms recorded during resting state, eyes-closed conditions characterized by quiet wakefulness, psychophysiological relaxation, and mind-wandering [39]. In cognitively healthy older adults, rsEEG alpha rhythms (8–12 Hz) predominate in the posterior scalp regions, while rsEEG delta (< 4 Hz) and theta (4–7 Hz) rhythms are typically widespread and low in amplitude [39]. Compared to Edu-Healthy individuals, Edu+ individuals exhibit higher posterior rsEEG alpha rhythms, supporting the neuroprotective hypothesis. In contrast, Edu+ patients with AD and mild cognitive impairment show more altered posterior rsEEG alpha rhythms, consistent with the compensatory hypothesis [40]. Similar findings have been observed in older adults with subjective memory complaints who are positive for AD biomarkers, compared to Edu- controls [40, 41].

The CR hypothesis has also been tested in PD and DLB patients, yielding mixed results [42–44]. In line with findings in AD, Edu+ PD and DLB patients generally exhibit better cognitive performance, particularly in executive functions and episodic memory, and experience less cognitive decline over time, taking disease symptom severity and duration into account [43, 45–53]. The strength of these associations varies depending on the clinical subtype of the disease, the CR measure used, and disease severity (for recent reviews on CR and alpha-synucleinopathy, see [54, 55]). However, it remains less clear than in AD whether PD and DLB Edu+ compared to Edu- patients may (1) maintain a similar cognitive status against greater brain neuropathology and neurodegeneration, in line with *compensatory mechanisms*, or (2) show better cognitive status and lower brain neuropathology and neurodegeneration, in line with a sort of *neuroprotective mechanisms acting even after the onset of dementia*.

According to the hypothesis of *compensatory (resilient) mechanisms*, a positive association between educational attainment, [(11)C] PiB PET-based amyloid deposition in the brain, and cognitive status was observed in PD patients with relatively low educational attainment, ranging from cognitively unimpaired to dementia [56]. This association was not found in patients with the highest educational attainment, suggesting a CR moderation effect [55]. In that study, the level of brain amyloidosis was similar in both PD subgroups, indicating that high CR in Edu+ patients did not prevent brain amyloidosis but merely mitigated its clinical effects [56]. Another study found a positive association between CR level (measured with a questionnaire), cognitive status, and [18 F]-flutemetamol-PET-based brain amyloidosis in PD patients with cognitive deficits [57].

Similar findings were reported in DLB patients. For instance, a negative association was found between educational attainment and metabolism in temporal, parietal, and occipital areas, as revealed by fluorine-18 fluorodeoxyglucose (FDG)-PET [58]. Another study reported a negative association between educational attainment and FDG-PET-based metabolism in occipital areas related to the degree of patient autonomy [59]. Additionally, there was a negative association between educational attainment and FDG-PET metabolism in the parietal cortex and precuneus in DLB patients [60]. Moreover, the DLB-typical diagnostic cingulate island sign was more prominent in patients with lower educational attainment [60]. Furthermore, a negative association was observed between specific occupational profiles (as CR proxies) and FDG-PET-based functional connectivity in the anterior default mode and visual cortical networks in DLB patients [52]. Finally, DLB patients with higher educational attainment exhibited stronger associations between clinical core features (e.g., parkinsonism, visual hallucinations, RBD, cognitive deficits) and specific patterns of brain hypometabolism and hypermetabolism [61].

In support of the hypothesis of *neuroprotective mechanisms even after the onset of cognitive deficits*, a positive association was reported between educational attainment and MRI-based gray matter volume in the basal ganglia of PD patients with cognitive deficits [62]. The same study also found a positive association between leisure time (another CR dimension) and gray matter volume in the dorsal prefrontal cortex [62]. Another study in PD patients with cognitive deficits reported a positive association between CR level (measured with a questionnaire), cognitive status, and FDG-PET metabolism in the right fusiform gyrus, lateral temporal, occipital, and parietal cortical areas [62]. In DLB patients, a positive association was observed between educational attainment, visual-cognitive functions, and dopamine transporter binding in the basal ganglia, as measured by 123-I-ioflupane single photon emission computed tomography (SPECT) [63].

Finally, previous studies explored the relationship between CR indices and functional connectivity in relevant brain networks. One study conducted in PD patients with cognitive deficits used resting-state functional MRI (rs-fMRI) to investigate functional brain connectivity [64]. Compared to PD Edu- patients, PD Edu+ patients exhibited greater functional connectivity in the anterior cingulate and basal ganglia, as well as bilaterally greater functional connectivity in frontoparietal regions within networks defined by dorsolateral and ventrolateral prefrontal seeds [64]. Another study was conducted on DLB patients using FDG-PET to investigate functional brain connectivity and found a positive association between educational attainment and FDG-PET functional connectivity in executive, attentive, and posterior default mode networks [52].

The present study aimed to investigate the effect of educational attainment on rsEEG rhythms in patients with PDD and DLB. This was done to evaluate the (neuroprotective or compensatory/resilient) impact of CR on the neurophysiological mechanisms underlying the regulation of cortical arousal and vigilance in quiet wakefulness, as revealed by those rhythms. This dimension has not been extensively explored, despite its significant clinical relevance. For instance, effective vigilance regulation is essential for activities such as reading newspapers, following TV programs, and engaging in social conversations with friends and relatives [65]. Prior research has demonstrated that individuals with PDD and DLB exhibit aberrantly large rsEEG delta and theta rhythms across a multitude of regions, accompanied by a reduction in posterior rsEEG alpha rhythms [66–74]. These alterations in rsEEG rhythms have also been associated with symptoms of vigilance dysfunction, such as visual hallucinations and RBD, in PDD and DLB patients [74, 75]. The neuroprotective effect of education was tested in a control group of cognitively unimpaired older adults. This study would help to elucidate the neuroprotective and compensatory role of education in relation to neurodegenerative disease progression. There is an urgent need to determine the extent to which socio-economic status is associated with educational inequalities in brain health and to develop appropriate strategies to compensate for these inequalities.

## Materials and methods

### Participants

The clinical and rsEEG datasets for the present investigation were sourced from the Eurasian archive of the PDWAVES Consortium (www.pdwaves.eu) and the European DLB Consortium. These datasets comprised records from 54 Healthy, 75 PDD, and 50 DLB participants, all of whom had undergone rsEEG recordings under the eyes-closed condition. Specifically, they were recruited by the following clinical units: the Sapienza University of Rome (Italy), the University “G. d’Annunzio” of Chieti-Pescara (Italy), the University of Salerno (Italy), the Institute for Research and Evidence-based Care (IRCCS) “Fatebenefratelli” of Brescia (Italy), the IRCCS Synlab SDN of Naples (Italy), the Oasi Research Institute-IRCCS, Troina (Italy), the IRCCS Ospedale Policlinico San Martino and DINOGMI (University of Genova, Italy), the Hospital San Raffaele of Cassino (Italy), the IRCCS San Raffaele Pisana of Rome (Italy), Newcastle University (UK), the Translational and Clinical Research Institute of Newcastle University (UK), Dokuz Eylül University, Faculty of Medicine (Izmir, Turkey), and Istanbul Medipol University (Turkey).

To assess the impact of educational attainment as a proxy of CR on the rsEEG activations, the enrolled Healthy, PDD, and DLB participants were stratified in sub-groups according to the median educational attainment of each group: Healthy-Edu- (*N* = 27; ≤ 10 years of educational attainment), Healthy-Edu+ (*N* = 27; > 10 years), PDD-Edu- (*N* = 30; < 10 years), PDD-Edu+ (*N* = 45; ≥ 10 years), DLB-Edu- (*N* = 21; < 10 years), and DLB-Edu+ (N = 29; ≥ 10 years). Within each group (i.e., Healthy, PDD, and DLB), the Edu- and Edu+ subgroups were matched for age, sex, and global cognitive status, ensuring that the groups shared identical mean values of age, education, and MMSE score.

The local institutional ethical committees approved the study. All experiments were conducted in accordance with the Code of Ethics of the World Medical Association (Declaration of Helsinki) and the standards established by the local institutional review boards. Informed and overt consent was obtained from each participant or caregiver prior to their participation.

### Diagnostic criteria

The diagnosis of PD (*N* = 75) was based on a standard clinical assessment of tremor, rigidity, and bradykinesia [76]. As measures of severity of a motor disability, the Hoehn and Yahr stage [77] and the Unified Parkinson Disease Rating Scale-III (UPDRS III [78]) for extrapyramidal symptoms were used (unfortunately, data from the Movement Disorder Society-Sponsored Revision of the Unified Parkinson’s Disease Rating Scale were not available). Furthermore, the diagnosis of PDD was given to patients with a history of dementia, preceded by a typical levodopa-responsive Parkinsonian motor syndrome for at least 12 months and unrelated to other pathologic conditions than PD.

The selected PDD patients did not suffer from severe tremors or dyskinesias. The PDD was diagnosed according to the Diagnostic and Statistical Manual of Mental Disorders criteria, fourth edition (DSM-IV-TR; American Psychiatric Association). The following inclusion criteria were fulfilled: (1) diagnosis of PD as specified previously; (2) a gradual neurocognitive decline in the context of established PD reported by either the patient or a reliable informant or observed by clinicians; (3) an abnormally low score in at least one of the neuropsychological tests mentioned in the following section, as defined by performances beyond 1.5 times the SD from the mean value for age- and education-matched controls or equivalent scores for abnormality according to the manuals of the tests used; and (4) moderate to severe impairment in instrumental activities of daily living and dependency. All PDD patients were under standard long-term chronic dopaminergic treatment, and all exams were performed under the ON state.

The diagnosis of the DLB (*N* = 50) was carried out according to the mentioned consensus guidelines [6]. The clinical features of the DLB patients were detected as follows: (1) the occurrence frequency and severity of hallucinations were evaluated using item-2 of the Neuropsychiatric Inventory (NPI [79]); (2) the severity of frontal dysfunction was assessed using the Frontal Assessment Battery (FAB [80]); (3) the presence and severity of the cognitive fluctuations were determined using the Clinician Assessment of Fluctuations [66, 67]; (4) extrapyramidal signs were evaluated using the Unified Parkinson Disease Rating Scale-III (UPDRS-III [78]); and (5) the presence and absence of RBD was determined based on the minimal International Classification of Sleep Disorders criteria (1992).

Exclusion criteria for healthy seniors were (1) neurological or psychiatric diseases (previous or present), (2) the presence of a depressive episode as detected by a Geriatric Depression Scale (15-item version) score higher than 5, (3) the use of chronic psychoactive drugs, and (4) the presence of significant chronic systemic illnesses such as diabetes mellitus.

In the study participants, the performance in various cognitive domains, including language, visuospatial function, executive function/attention, and memory, was assessed using local batteries of neuropsychological tests. Specifically, for the evaluation of cognitive functions, (1) language was tested by the verbal fluency test for letters [81] and the verbal fluency test category (fruits, animals, or car trades [81]); (2) visuospatial functions were assessed by the line orientation test [82] and the face recognition test [83]; (3) executive functions and attention were evaluated by the trail-making test parts A and B [84], the Stroop test [85], and the Confusion Assessment Method (executive function part [86]); and (4) memory was tested by the digit span forward and backward [87], Oktem verbal memory test [88], and the Confusion Assessment Method (memory part [86]). Notably, each clinical unit administered one or more of the above-mentioned neuropsychological tests for each cognitive domain, according to the local protocols.

### The rsEEG recordings

The rsEEG recordings were conducted using local routine professional digital EEG systems licensed for clinical applications (we used the equipment brands as a covariate in the statistical models of the present study). All rsEEG recordings were performed in the morning to minimize potential variations due to circadian rhythms. Standard instructions for the resting-state condition emphasized staying awake, psychophysically relaxed with mind wandering, and following the experimenter’s requests to keep the eyes closed and open during the rsEEG recording. The experiments checked the participant’s behavioral state during the EEG recordings and annotated eventual deviations and alarms.

**Fig. 1:**
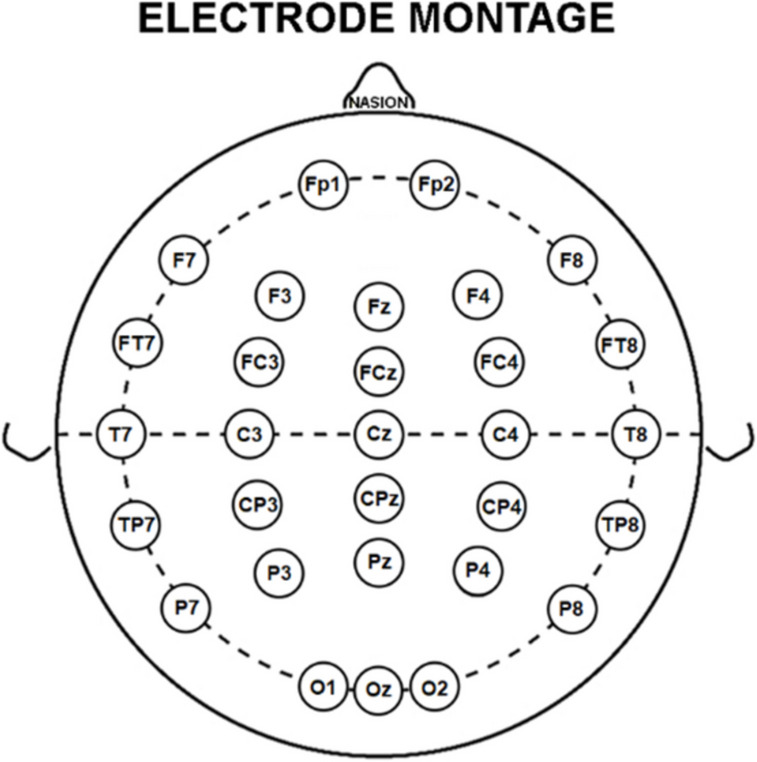
Electroencephalographic (EEG) electrode montage. The electrode montage included 30 scalp monopolar sensors placed following the 10–10 system (i.e., Fp1, Fp2, F7, F3, Fz, F4, F8, FT7, FC3, FCz, FC4, FT8, T7, C3, Cz, C4, T8, TP7, CP3, CPz, CP4, TP8, P7, P3, Pz, P4, P8, O1, Oz and O2). This montage was used to record the resting-state EEG (rsEEG) activity during the eyes-closed condition

A common electrode montage of 30 scalp exploring electrodes, placed according to the 10–10 system, characterized the EEG data recordings in all clinical units, and was used for the data analysis. These electrodes, referred to as “selected electrodes,” were positioned to cover the whole scalp (Fig. 1). The reference electrode was typically placed between Fz and Cz of the 10–20 system, and the ground electrode was in the posterior midline. To minimize the influence of the different placement of the reference electrodes, all EEG data were re-referenced to the common average for the data analysis. Electrooculographic (EOG) activity with a standard bipolar montage was also recorded to monitor and control eye movements and blinking. As minimum standards in all clinical units, electrophysiological data allowed a bandpass filtering of 0.3–70 Hz and a sampling rate of 256 Hz.

### Preliminary rsEEG data analysis

The rsEEG data were centrally analyzed by experts blinded to the participants’ diagnosis by the Sapienza University of Rome unit, who were blinded to the participant’s diagnosis. The recorded rsEEG data were exported as a European data format (.edf) or EEGLAB set (.set) files and then processed offline using the EEGLAB toolbox ([89]; version eeglab14_1_2b) running in the MATLAB software (Mathworks, Natick, MA, USA; version: R2014b).

During the preprocessing, the rsEEG data were divided into epochs lasting 2 s (i.e., 5 min = 150 rsEEG epochs of 2 s for each experimental condition) and subjected to offline analysis. A three-step procedure was employed to detect and remove the following items: (1) recording channels (electrodes) showing prolonged artifactual rsEEG activity due to bad electric contacts or other reasons; (2) rsEEG epochs with artifacts at recording channels characterized by generally good signals; and (3) intrinsic components of the rsEEG epochs with artifacts.

The first step involved a visual analysis of the recorded rsEEG activity by two independent experimenters chosen from a panel of four experts (i.e., S.L., C.D.P, R.L., and G.N). Their task was to identify electrodes affected by irremediable artifacts. Typically, up to three selected electrodes were removed for each participant. In cases where the clinical units utilized a digital EEG system with more than 30 exploring electrodes, the removed electrodes were substituted with the nearest electrodes not included among the 30 selected electrodes. These added electrodes were then used alongside the artifact-free selected electrodes to compute the interpolation of artifact-free rsEEG data at all scalp sites corresponding to the removed electrodes. This process, facilitated by the EEGLAB toolbox ([89]; version eeglab14_1_2b), ensured that all participants had artifact-free EEG data at the locations of the 30 selected electrodes.

The second step involved a visual analysis of the recorded rsEEG activity by the experimenters (i.e., S.L., C.D.P, R.L., and G.N.) to identify artifactual rsEEG epochs. These epochs were contaminated by muscular, ocular, head movements, or non-physiological artifacts and were subsequently removed from the database. Notably, muscle tension artifacts were identified through careful observation of the effects of various frequency bandpass filters and inspection of EEG power density spectra. These artifacts manifested as unusually high and stable values of EEG power density in the frequency range of 30 to 70 Hz, contrasting with the typical declining trend observed in EEG power density from 25 Hz onward.

The third step involved implementing independent component analysis (ICA) using the EEGLAB toolbox to remove components representing residual artifacts. These artifacts included (1) blinking and eye movements, (2) involuntary head movements, (3) neck and shoulder muscle tensions, and (4) electrocardiographic pulse activity. For each rsEEG dataset, less than 5 ICA components were removed from the original solutions, which were based on 30 ICA components. Subsequently, the rsEEG datasets were reconstructed with the remaining artifact-free ICA components. To ensure the integrity of the data, the putative artifact-free rsEEG epochs underwent visual double-checking by the independent experimenters (i.e., S.L., C.D.P., R.L., and G.N.). This step confirmed or made the final decision about the inclusion or exclusion of each rsEEG epoch.

For harmonization purposes, the artifact-free EEG data for the common 30 electrodes were digitally frequency-band passed at 0.1–45 Hz. They also were down-sampled, when appropriate, to make the sampling rate of all artifact-free rsEEG datasets equal to 256 Hz. Furthermore, the EEG data were re-referenced to the common average reference.

As a result of the above procedures, the artifact-free epochs showed a similar proportion (greater than 75%) of the total amount of rsEEG activity recorded in all groups of participants (i.e., Healthy, PDD, and DLB).

### Spectral analysis of the rsEEG epochs

Standard digital fast Fourier Transform (FFT)–based analysis, employing the Welch technique with a Hanning windowing function and no phase shift, was used to compute the power density of the artifact-free rsEEG epochs recorded at all 30 scalp electrodes, with a frequency resolution of 0.5 Hz.

The rsEEG frequency bands of interest were individually identified based on specific frequency landmarks, namely the transition frequency (TF) and the individual alpha frequency (IAF). In the (eyes-closed) rsEEG power density spectrum, the TF was defined as the minimum rsEEG power density between 3 and 8 Hz. In comparison, the IAF peak was defined as the maximum power density peak between 6 and 14 Hz.

The TF and IAF were computed for each participant involved in the study. Based on TF and IAF, individual delta, theta, and alpha bands were estimated as follows: delta from TF − 4 Hz to TF − 2 Hz, theta from TF − 2 Hz to TF, low alpha (alpha 1 and alpha 2) from TF to IAF, and high-frequency alpha (or alpha 3) from IAF to IAF + 2 Hz. Specifically, the individual alpha 1 and alpha 2 bands were computed as follows: alpha 1 from TF to the frequency midpoint of the TF–IAF range and alpha 2 from that midpoint to the IAF peak. The other bands were defined based on standard fixed frequency ranges used in the reference rsEEG studies of our Consortium [40, 41]: beta 1 from 14 to 20 Hz, beta 2 from 20 to 30 Hz, and gamma from 30 to 40 Hz.

### Estimation of rsEEG source activation

We used the official freeware tool called exact LORETA (eLORETA) to linearly estimate the cortical source activity generating scalp-recorded rsEEG rhythms). The present implementation of the eLORETA freeware uses a head volume conductor model composed of the scalp, skull, and cerebral cortex. In the scalp compartment, the exploring electrodes can be virtually positioned to give EEG data as an input to the source estimation [90]. The cortical model is based on a realistic cerebral shape from a template typically used in neuroimaging studies, namely that of the Montreal Neurological Institute (MNI152 template). Using eLORETA, the EEG inverse problem is solved from scalp-recorded EEG activity, estimating the associated “neural” current density values at any cortical voxel within the head volume conductor model. Cortical source solutions are computed at each voxel of the model and each frequency bin.

For the estimation of EEG cortical source activities (i.e., the eLORETA solutions), the input consists of EEG spectral power density computed at the 30 scalp electrodes. This estimation occurs within the electrical cortical source space, which comprises 6239 voxels with 5 mm resolution, restricted to the cortical grey matter of the head volume conductor model. Within each voxel, an equivalent current dipole is located to represent the mean ionic currents generated by the local populations of cortical pyramidal neurons. The eLORETA package provides the Talairach coordinates, the lobe, and the Brodmann area (BA) for each voxel of the cortical model.

The eLORETA solutions from the rsEEG data were normalized by the following procedure. We averaged the eLORETA solutions across (1) all frequency bins from 0.5 to 45 Hz and (2) 6239 voxels of the cortical model to obtain the eLORETA “mean” solution. Afterward, we computed the ratio between any original eLORETA solution at a given condition/frequency-bin/voxel and the eLORETA mean solution. As a result, any original eLORETA solution at a given condition/frequency-bin/voxel changed to a normalized eLORETA solution.

In line with the general low spatial resolution of the current EEG methodological approach (i.e., 30 scalp electrodes), we performed a regional analysis of the eLORETA solutions. For this purpose, we separately collapsed the eLORETA solutions within frontal (BAs 8, 9, 10, 11, 44, 45, 46, and 47), central (BAs 1, 2, 3, 4, and 6), parietal (BAs 5, 7, 30, 39, 40, and 43), occipital (BAs 17, 18, and 19), temporal (BAs 20, 21, 22, 37, 38, 41, and 42), and limbic (BAs 31, 32, 33, 34, 35, and 36) macro-regions (ROIs).

For the present eLORETA cortical source estimation, a frequency resolution of 0.5 Hz was used.

### Main statistical analysis of rsEEG source activities: Healthy-Edu- vs. Healthy-Edu+, PDD-Edu- vs. PDD-Edu+, and DLB- Edu- vs. DLB-Edu+ 

A main statistical analysis was conducted using the commercial software STATISTICA 10 (StatSoft Inc., www.statsoft.com) to test the working hypothesis that the rsEEG source activities may be related to the sex in the Healthy, PDD, and DLB groups.

Three separate ANOVAs were performed (one for each group), with rsEEG source activities (i.e., regional normalized eLORETA solutions) as a dependent variable (*p* < 0.05). The ANOVAs included the factors Education (Edu- and Edu+, independent variable), Band (delta, theta, alpha 1, alpha 2, alpha 3, beta 1, beta 2, and gamma), and ROI (frontal, central, parietal, occipital, temporal, and limbic). The sex, the global cognition (MMSE), and the clinical unit were used as covariates. Post hoc comparisons were conducted using the Duncan test (*p* < 0.05).

Confirmation of the working hypothesis required two criteria to be satisfied: (1) a statistically significant ANOVA interaction involving the Education factor (*p* < 0.05) and (2) a post-hoc Duncan test revealing statistically significant (*p* < 0.05 Bonferroni corrected) differences in the rsEEG source activities between the Edu- and Edu+ subgroups.

The results of the main statistical analysis were subjected to the Grubbs test to evaluate the eventual presence of outliers (*p* < 0.001).

For cross-validation purposes, the same experimental designs were applied to a separate experimental cohort extracted from a previous database of rsEEG data. The participants were enrolled in the same clinical units with the procedure described in the previous sections. The rsEEG data were acquired with a 19-electrode montage (10–20 international system) and the same recording protocol (eyes closed and open conditions). The same rsEEG preprocessing and source activity estimation described above were applied. To reach a satisfying sample, we included patients both at the prodromal (mild cognitive impairment, MCI) and dementia stages for PD and LB diseases. The following participants were included: Healthy (*n* = 54), PD (MCI and dementia; N = 38), and DLB (MCI and dementia; *N* = 44). In the Supplementary Materials Methods, we included details concerning the diagnostic procedure for MCI definition in this separate cohort. The same statistical designs were applied to evaluate the effect of educational attainment on rsEEG source activities in Healthy, PD (MCI and dementia), and DLB (MCI and dementia).

## Results

### Demographic, clinical, and rsEEG source markers in Healthy-Edu- and Healthy-Edu+ subgroups

Table 1 summarizes the relevant demographic (i.e., age, education, and sex) and clinical (i.e., MMSE score raw and corrected for age and education) information about the Healthy-Edu- (*N* = 27) and Healthy-Edu+ (*N* = 27) subgroups, together with the results of the statistical analyses computed to evaluate the presence or absence of statistically significant differences between these subgroups regarding age (*T*-test), sex (Fisher test), education (*T*-test), and MMSE score (raw and corrected; Mann-Whitney *U* test). As expected, statistically significant differences were found between the Healthy-Edu- and the Healthy-Edu+ subgroups for educational attainment (*p* < 0.01). On the contrary, no statistically significant differences in age, sex, and MMSE score (raw and corrected) were found between the two subgroups (*p* > 0.05).

**Table 1:** Mean values (± standard error of the mean, SE) of the demographic and clinical data as well as the results of their statistical comparisons (*p* < 0.05) in the subgroups of Healthy-Edu- (*N* = 27) and Healthy-Edu+ (*N* = 27) participants. Each group was partitioned into low (Edu-) and high (Edu+) educational attainment subgroups, matched for age, sex, and cognitive-motor status. Legend: *Healthy*, cognitively unimpaired older persons; *M/F*, males/females; *MMSE*, Mini*-*Mental State Evaluation; *MMSEc*, Mini-Mental State Evaluation corrected for age and educational attainment; *n.s.*, not significant (*p* > 0.05)

Demographic and clinical data			
	Healthy-Edu-	Healthy-Edu+	Statistical analyses
*N*	27	27	**–**
Age (years)	68.3 ± 1.5 SE	70.0 ± 1.7 SE	*T*-test: n.s
Sex (M/F)	11/16 (41%)	14/13 (52%)	Fisher test: n.s
Education (years)	7.0 ± 0.6 SE	14.0 ± 0.5 SE	*T*-test: *p* < 0.01
MMSE score	28.4 ± 0.4 SE	28.6 ± 0.4 SE	Mann Whitney *U* test: n.s
MMSEc score	27.8 ± 0.3 SE	27.3 ± 0.3 SE	Mann Whitney *U* test: n.s

The mean TF was 5.6 Hz (± 0.2 SE) in the Healthy-Edu- subgroup and 5.8 Hz (± 0.2 SE) in the Healthy-Edu+ subgroup. Furthermore, the mean IAF was 8.9 Hz (± 0.2 SE) in the Healthy-Edu- subgroup and 9.0 Hz (± 0.2 SE) in the Healthy-Edu+ subgroup. Two *T*-tests (p < 0.05) were performed to evaluate the presence or absence of statistically significant differences between the Healthy-Edu- and the Healthy-Edu+ subgroups regarding TF and IAF. No statistically significant differences were found between the two subgroups (*p* > 0.05).

**Fig. 2:**
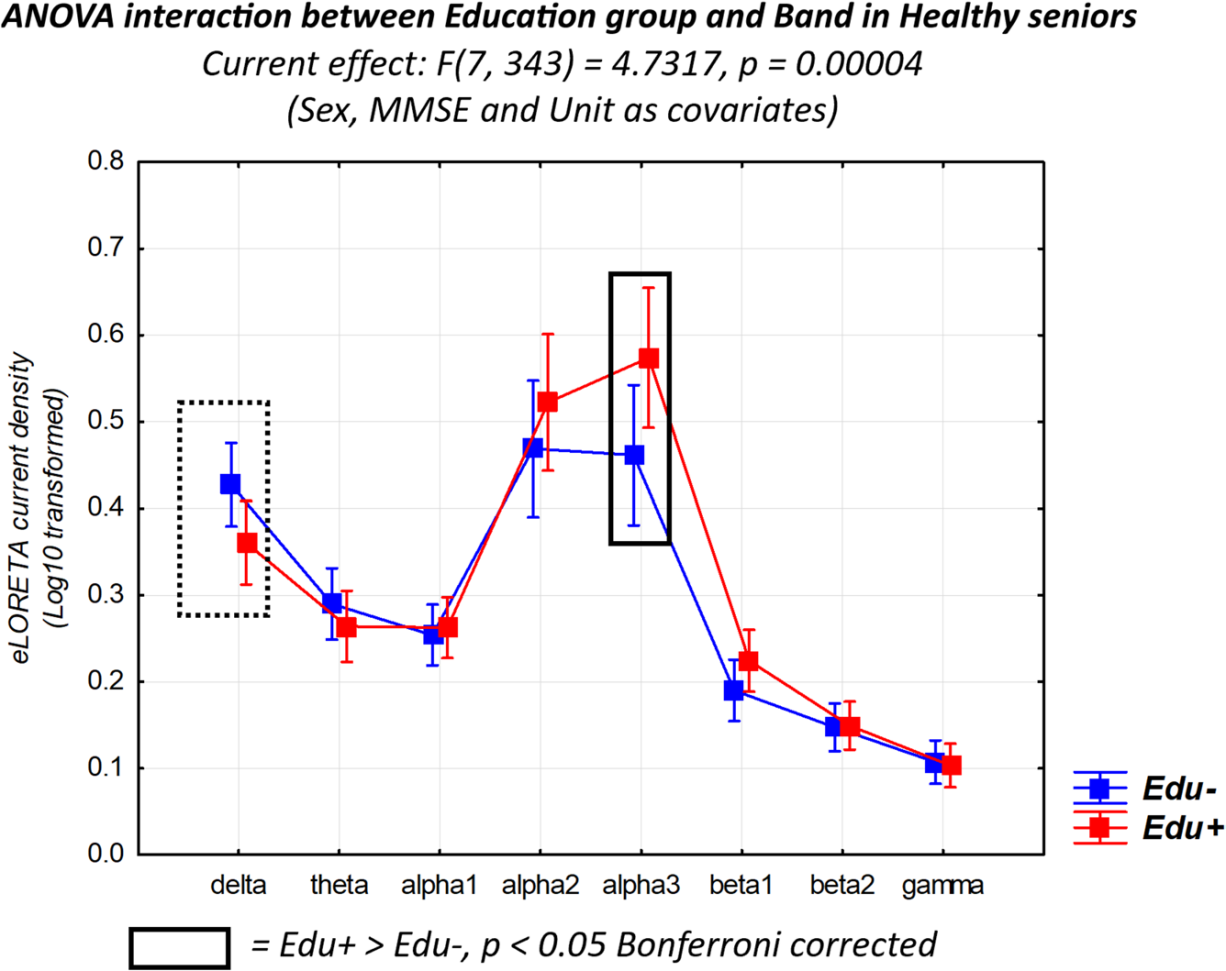
Mean values (± standard error of the mean SE, Log-10 transformed) of rsEEG source activities (normalized eLORETA current density) relative to a statistically significant ANOVA interaction effect (*F* = 4.73, *p* < 0.01) between the factors Education (Healthy-Edu-, *N* = 27; and Healthy-Edu+, *N* = 27) and Band (delta, theta, alpha 1, alpha 2, alpha 3, beta 1, beta 2, and gamma). The sex, MMSE, and clinical unit were used as a covariate. The rectangles indicate the frequency bands in which the eLORETA solutions statistically presented a significant difference between Healthy-Edu- and Healthy-Edu+ subgroups (*p* < 0.05 Bonferroni corrected). Legend: *Healthy*, cognitively unimpaired older persons; *rsEEG*, resting-state electroencephalographic

Figure 2 shows the mean values (± SE, log-10 transformed) of rsEEG source activities (normalized eLORETA current density) relative to a statistically significant two-way ANOVA interaction effect (F(7, 343) = 4.73, *p* < 0.01) between the factors Education (Healthy-Edu- and Healthy-Edu+; dependent variable) and Band (delta, theta, alpha 1, alpha 2, alpha 3, beta 1, beta 2, and gamma). The effect size was quantified by h_p_^2^ = 0.09 (medium to large effect size). The Duncan planned post hoc (p Healthy-Edu- was fitted by the rsEEG alpha 3 (p 0.05).

The findings mentioned above were not due to outliers from individual normalized eLORETA current densities (Log10 transformed), as shown by Grubbs’ test with an arbitrary threshold of *p* > 0.001 (See Supplementary Materials Figure SM1).

### Demographic, clinical, and rsEEG source markers in PDD-Edu- and PDD-Edu+ subgroups

Table 2 summarizes the relevant demographic (i.e., age, education, and sex) and clinical (i.e., MMSE score raw and corrected for age and education, visual hallucination, VH, and Unified Parkinson Disease Rating Scale-III, UPDRS III score; REM behavioral disorder, RBD) information about the PDD-Edu- (*N* = 30) and PDD-Edu+ (*N* = 45) subgroups, together with the results of the statistical analyses computed to evaluate the presence or absence of statistically significant differences between these subgroups regarding age (*T*-test), sex (Fisher test), education (*T*-test), MMSE score (raw and corrected; Mann-Whitney *U* test), VH (Fisher test), UPDRS III score (*T*-test). As expected, statistically significant differences were found between the PDD-Edu- and PDD-Edu+ subgroups for educational attainment (*p* < 0.01). The MMSE score was higher in the PDD-Edu+ subgroup than in the PDD-Edu- subgroup (*p* < 0.01). On the contrary, no statistically significant differences in age, sex, MMSE score corrected, VH, UPDRS III score, and RBD were found between the two subgroups (*p* > 0.05).

**Table 2:** Mean values (± SE) of the demographic and clinical data as well as the results of their statistical comparisons (*p* < 0.05) in the subgroups of PDD-Edu- (*N* = 30) and PDD-Edu+ (*N* = 45) participants. Each group was partitioned into low (Edu-) and high (Edu+) educational attainment subgroups, matched for age, sex, and cognitive-motor status. Legend: *PDD*, Parkinson’s disease dementia; *M/F*, males/females; *MMSE*, Mini-Mental State Evaluation; *MMSEc*, Mini-Mental State Evaluation corrected for age and educational attainment; *VH*, visual hallucination; *UPDRS III*, Unified Parkinson Disease Rating Scale-III; *RBD*, REM behavioral disorder; *n.s.*, not significant (*p* > 0.05)

Demographic and clinical data			
	PDD-Edu-	PDD-Edu+	Statistical analyses
N	30	45	-
Age (years)	71.5 ± 1.4 SE	73.3 ± 0.8 SE	*T*-test: n.s
Sex (M/F)	25/5 (83%)	37/8 (82%)	Fisher test: n.s
Education (years)	3.6 ± 0.4 SE	10.9 ± 0.2 SE	*T*-test: *p* < 0.01
MMSE score	18.1 ± 0.7 SE	21.0 ± 0.7 SE	Mann Whitney *U* test: *p* < 0.01
MMSEc score	18.1 ± 0.7 SE	19.8 ± 0.7 SE	Mann Whitney *U* test: n.s
VH (%)	53%	56%	Fisher test: n.s
UPDRS III	39.6 ± 3.8 SE	41.3 ± 3.0 SE	*T*-test: n.s
RBD (%)	53%	53%	Fisher test: n.s
Disease duration (years)	8.3 ± 1.4 SE	3.6 ± 0.8 SE	*T*-test: p < 0.01

The mean TF was 4.5 Hz (± 0.1 SE) in the PDD-Edu- subgroup and 4.7 Hz (± 0.1 SE) in the PDD-Edu+ subgroup. Furthermore, the mean IAF was 7.2 Hz (± 0.2 SE) in the PDD-Edu- subgroup and 6.7 Hz (± 0.1 SE) in the PDD-Edu+ subgroup. Two *T*-tests (*p* < 0.05) were performed to evaluate the presence or absence of statistically significant differences between the PDD-Edu- and PDD-Edu+ subgroups regarding TF and IAF. No statistically significant differences were found between the two subgroups (*p* > 0.05).

Table 3 illustrates the main pharmacological therapies administered to the PDD-Edu- and PDD-Edu+ subgroups, together with the results of the statistical analyses computed to evaluate the presence or absence of statistically significant differences between these subgroups regarding the percentage of patients undergoing to a specific pharmacological treatment (Fisher test) and regarding the levodopa equivalent dose (LED, mg, *T*-test). No statistically significant differences were found between the PDD-Edu- and PDD-Edu+ subgroups for any pharmacological treatment nor LED (*p* > 0.05).

**Table 3:** Pharmacological therapies administered to the PDD-Edu- and PDD-Edu+ subgroups. Legend: *PDD*, Parkinson’s disease dementia; *n.s.*, not significant (*p* > 0.05)

Drugs	PDD-Edu-		PDD-Edu+		Statistical analysis
	*N*	%	*N*	%	
High blood pressure drugs	3	10%	6	13%	Fisher test = n.s
Diabetes drugs	2	7%	6	13%	Fisher test = n.s
Selective serotonin reuptake inhibitors (SSRIs)	5	17%	4	9%	Fisher test = n.s
Anxiolytics	0	0%	3	7%	Fisher test = n.s
Antipsychotics	4	13%	4	16%	Fisher test = n.s
Antagonists of N-methyl-d-aspartate receptors (aNMDARs)	6	20%	5	11%	Fisher test = n.s
Acetylcholinesterase inhibitors (AChEIs)	7	23%	16	36%	Fisher test = n.s
Antiparkinsonian drugs (levodopa)	29	97%	44	98%	Fisher test = n.s
	**Mean ± SE**		**Mean ± SE**		
Levodopa equivalent dose (LED; mg)	608.3 ± 76.0		606.3 ± 48.3		*T*-test = n.s

**Fig. 3:**
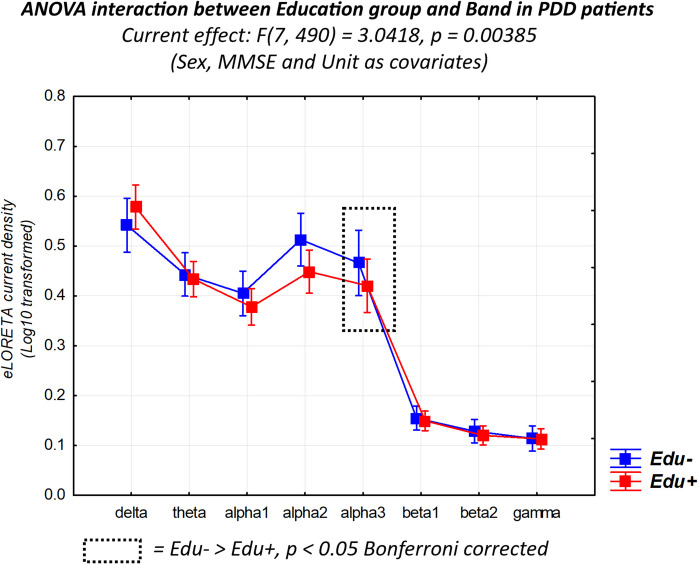
Mean values (± SE, Log-10 transformed) of rsEEG source activities (normalized eLORETA current density) relative to a statistically significant ANOVA interaction effect (*F* = 3.04, *p* < 0.01) between the factors Education (PDD-Edu-, *N* = 30, and PDD-Edu+, *N* = 45) and Band (delta, theta, alpha 1, alpha 2, alpha 3, beta 1, beta 2, and gamma). The sex, MMSE, and clinical unit were used as a covariate. The rectangles indicate the frequency bands in which the eLORETA solutions statistically presented a significant difference between PDD-Edu- and PDD–Edu+ (*p* < 0.05 Bonferroni corrected). Legend: *PDD*, Parkinson’s disease dementia; *rsEEG*, resting-state electroencephalographic

Figure 3 shows the mean values (± SE, log-10 transformed) of rsEEG source activities (normalized eLORETA current density) relative to a statistically significant two-way ANOVA interaction effect (*F*(7, 490) = 3.05, *p* < 0.01) between the factors Education (PDD-Edu- and PDD-Edu+; dependent variable) and Band (delta, theta, alpha 1, alpha 2, alpha 3, beta 1, beta 2, and gamma). The effect size was quantified by η_p_^2^ = 0.09 (medium to large effect size). The Duncan planned post hoc (*p* < 0.05 Bonferroni correction for 8 frequency bands, *p* < 0.05/8 = 0.006) testing showed that the discriminant pattern PDD-Edu- > PDD-Edu+ was fitted by the rsEEG alpha 3 (*p* < 0.05 Bonferroni corrected) source activity. No other effects involving the factor of Education were observed (*p* > 0.05).

The findings mentioned above were not due to outliers from individual normalized eLORETA current densities (Log10 transformed), as shown by Grubbs’ test with an arbitrary threshold of *p* > 0.001 (See Supplementary Materials Figure SM2).

### Demographic, clinical, and rsEEG source markers in DLB-Edu- and DLB-Edu+ subgroups

Table 4 summarizes the relevant demographic (i.e., age, education, and sex) and clinical (i.e., MMSE score raw and corrected for age and education, visual hallucination, VH, and Unified Parkinson Disease Rating Scale-III, UPDRS III score; REM behavioral disorder, RBD) information about the DLB-Edu- (*N* = 21) and DLB-Edu+ (*N* = 29) subgroups, age (*T*-test), sex (Fisher test), education (*T*-test), MMSE score (raw and corrected; Mann-Whitney *U* test), VH (Fisher test), and UPDRS III score (*T*-test). As expected, statistically significant differences were found between the DLB-Edu- and DLB-Edu+ subgroups for educational attainment (*p* < 0.01). On the contrary, no statistically significant differences in age, sex, MMSE score (raw and corrected), VH, UPDRS III score, and RBD were found between the two subgroups (*p* > 0.05).

**Table 4:** Mean values (± SE) of the demographic and clinical data as well as the results of their statistical comparisons (p < 0.05) in the subgroups of DLB-Edu- (*N* = 21) and DLB-Edu+ (*N* = 29) participants. Each group was partitioned into low (Edu-) and high (Edu+) educational attainment subgroups, matched for age, sex, and cognitive-motor status. Legend: *DLB*, dementia due to Lewy body disease; *M/F*, males/females; *MMSE*, Mini-Mental State Evaluation; *MMSEc*, Mini-Mental State Evaluation corrected for age and educational attainment; *VH*, visual hallucination; *UPDRS III*, Unified Parkinson Disease Rating Scale*-*III; *RBD*, REM behavioral disorder; *n.s.*, not significant (*p* > 0.05)

Demographic and clinical data			
	DLB-Edu-	DLB-Edu+	Statistical analyses
*N*	21	29	-
Age (years)	74.0 ± 1.8 SE	75.1 ± 1.6 SE	*T*-test: n.s
Sex (M/F)	12/9 (41%)	23/6 (79%)	Fisher test: n.s
Education (years)	5.0 ± 0.5 SE	11.5 ± 1.3 SE	*T*-test: *p* < 0.01
MMSE score	19.1 ± 1.3 SE	21.6 ± 0.8 SE	Mann Whitney *U* test: n.s
MMSEc score	18.8 ± 1.3 SE	20.2 ± 0.9 SE	Mann Whitney *U* test: n.s
VH (%)	76%	62%	Fisher test: n.s
UPDRS III	13.5 ± 1.6 SE	15.8 ± 1.4 SE	*T*-test: n.s
RBD (%)	75%	57%	Fisher test: n.s
Disease duration (years)	1.1 ± 0.4 SE	2.3 ± 0.4 SE	*T*-test: *p* < 0.05

The mean TF was 4.7 Hz (± 0.2 SE) in the DLB-Edu- subgroup and 4.8 Hz (± 0.2 SE) in the DLB-Edu+ subgroup. Furthermore, the mean IAF was 7.6 Hz (± 0.3 SE) in the DLB-Edu- subgroup and 7.0 Hz (± 0.2 SE) in the DLB-Edu+ subgroup. Two *T*-tests (*p* < 0.05) were performed to evaluate the presence or absence of statistically significant differences between the DLB-Edu- and DLB-Edu+ subgroups regarding TF and IAF. No statistically significant differences were found between the two subgroups (*p* > 0.05).

Table 5 illustrates the main pharmacological therapies administered to the DLB-Edu- and DLB-Edu+ subgroups, together with the results of the statistical analyses computed to evaluate the presence or absence of statistically significant differences between these subgroups regarding the percentage of patients undergoing to a specific pharmacological treatment (Fisher test) and regarding the levodopa equivalent dose (LED, mg, *T*-test). No statistically significant differences were found between the DLB-Edu- and DLB-Edu+ subgroups for any pharmacological treatment (*p* > 0.05). On the contrary, a statistically significant difference was found between the two subgroups for LED (*p* < 0.01). The LED was higher in the DLB-Edu- subgroup than in the DLB-Edu+ subgroup (*p* < 0.01). Due to this difference, the LED was included as a covariate in the following statistical analysis.

**Table 5:** Pharmacological therapies administered to the DLB-Edu- and DLB-Edu+ subgroups. Legend: *DLB*, dementia due to Lewy body disease; *n.s.*, not significant (*p* > 0.05)

Drugs	DLB-Edu-		DLB-Edu+		Statistical analysis
	*N*	%	*N*	%	
High blood pressure drugs	6	29%	12	41%	Fisher test = n.s
Diabetes drugs	8	38%	12	41%	Fisher test = n.s
Selective serotonin reuptake inhibitors (SSRIs)	10	48%	8	28%	Fisher test = n.s
Anxiolytics	0	0%	1	3%	Fisher test = n.s
Antipsychotics	5	24%	3	10%	Fisher test = n.s
Antagonists of N-methyl-d-aspartate receptors (aNMDARs)	2	10%	1	3%	Fisher test = n.s
Acetylcholinesterase inhibitors (AChEIs)	16	76%	26	90%	Fisher test = n.s
Antiparkinsonian drugs (levodopa)	11	52%	13	45%	Fisher test = n.s
	**Mean ± SE**		**Mean ± SE**		
Levodopa equivalent dose (LED; mg)	261.5 ± 38.0		153.5 ± 22.0		T test = p < 0.01

The ANOVA did not show any statistically significant three- or two-way interaction effect (*p* > 0.05) between the factors Education (DLB-Edu- and DLB-Edu+; dependent variable), Band (delta, theta, alpha 1, alpha 2, alpha 3, beta 1, beta 2, and gamma), and ROI (frontal, central, parietal, occipital, temporal, and limbic).

The findings mentioned above were not due to outliers from individual normalized eLORETA current densities (log 10 transformed), as shown by Grubbs’ test with an arbitrary threshold of *p* > 0.001 (See Supplementary Materials Figure SM3).

**Fig. 4:**
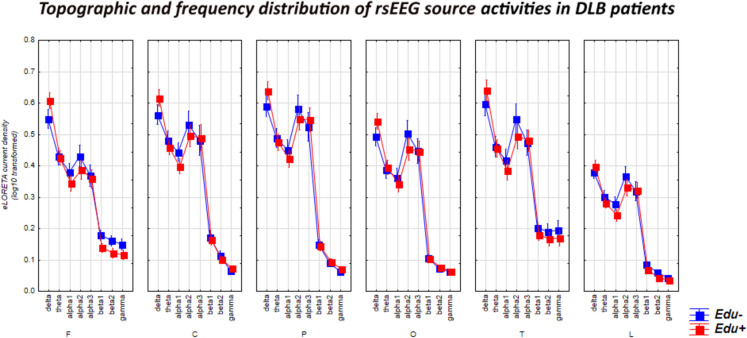
Mean values (± standard error of the mean SE, Log-10 transformed) of rsEEG source activities (normalized eLORETA current density) in DLB participants according to the factors Education (DLB-Edu- and DLB-Edu+; dependent variable), Band (delta, theta, alpha 1, alpha 2, alpha 3, beta 1, beta 2, and gamma), and ROI (frontal, central, parietal, occipital, temporal, and limbic). The correspondent ANOVA was not statistically significant for the 3-way interaction among the factors Education, Band, and ROI (*p* > 0.05). The sex, clinical unit, and LED were used as a covariate. Legend: *DLB*, dementia due to Lewy body disease; *rsEEG*, resting state electroencephalographic; *ROI*, region of interest

For illustrative purposes, Figure 4 shows the mean values (± SE, log-10 transformed) of rsEEG source activities (normalized eLORETA current density) in relation to the factors Education (DLB-Edu- and DLB-Edu+), Band (delta, theta, alpha 1, alpha 2, alpha 3, beta 1, beta 2, and gamma), and ROI (frontal, central, parietal, occipital, temporal, and limbic). Very similar rsEEG alpha source activities were observed between the DLB-Edu- and DLB-Edu+ groups (p > 0.05). Furthermore, figures reported in the Supplementary Materials illustrate the topographic and frequency distribution of the rsEEG alpha 2 and alpha 3 source activities for all the Healthy (Figure SM4) and PDD (Figure SM5) Edu- and Edu+ subgroups.

### Control analysis on the effect of participants’ linguistic-cultural background

A control analysis tested the effect of the participants’ linguistic-cultural background as a factor on the relationship between educational attainment and rsEEG rhythms in the Healthy, DLB, and PDD groups. Based on the limited composition of the available cohort, we could only form two sub-groups of participants for that factor, namely European (Italy and the UK) and Turkish. Specifically, for each group of participants (Healthy, DLB, and PDD), the control ANOVA (*p* < 0.05) included the factors of Linguistic-cultural background (Europe and Turkey), Education (Edu- and Edu+), Band (delta, theta, alpha 1, alpha 2, alpha 3, beta 1, beta 2, gamma), and ROI (frontal, central, parietal, occipital, temporal, and limbic). Results showed no effect of the linguistic-cultural background factor (*p* > 0.05). Furthermore, they confirmed a two-way interaction (*p* > 0.05) between Education (Edu-, Edu+) and Band (delta, theta, alpha 1, alpha 2, alpha 3, beta 1, beta 2, gamma) for all the groups as in the core findings (i.e., Healthy, DLB, and PDD groups). We reported the details of this control analysis in the Supplementary Materials (Figure SM6).

### Control analysis matching the educational attainment threshold defining the low and high-education sub-groups within the Healthy, DLB, and PDD participants

A control analysis (*p* < 0.05) tested the effect of possible bias due to the different thresholds used to stratify the low (Edu-) and high (Edu+) education subgroups in the Healthy, DLB, and PDD groups of the main statistical analysis. For this purpose, this analysis matched the educational attainment levels of the Edu- and Edu+ subgroups across the Healthy, DLB, and PDD participants. For each subgroup of participants (Healthy, DLB, and PDD), the control ANOVA (*p* < 0.05) included the factors of Education (Edu- and Edu+), Band (delta, theta, alpha 1, alpha 2, alpha 3, beta 1, beta 2, gamma), and ROI (frontal, central, parietal, occipital, temporal, and limbic). Results confirmed those obtained on the whole cohort, showing that the discriminant pattern Healthy–Edu+  > Healthy-Edu- was fitted by the rsEEG alpha 3 (*p* < 0.05 Bonferroni corrected) source activities. On the contrary, the discriminant pattern PDD-Edu- > PDD–Edu+ was fitted by the rsEEG alpha 2 and alpha 3 (*p* < 0.05 Bonferroni corrected) source activities. No statistically significant effects were observed for the DLB group (*p* > 0.05). We reported the details of this control analysis in the Supplementary Materials (Figure SM7).

### Association among CR, rsEEG alpha 2 and alpha 3 source activities, and cognitive scores in Healthy, PDD, and DLB patients

The main ANOVA results showed that rsEEG alpha 2 and alpha 3 source activities were affected by the education attainment in the Healthy participants and the PDD patients. Furthermore, they showed that these alpha source activities were greater in the Healthy-Edu+ subgroup than in the Healthy-Edu- subgroup, possibly because of the effect of neurophysiological neuroprotective mechanisms. In contrast, these alpha source activities were lower in the PDD-Edu+ subgroup than in the PDD-Edu- subgroup, possibly because of the effect of compensatory mechanisms. To control for these findings and explore the relationship between the rsEEG alpha source activity and cognitive functions in Healthy, DLB, and PDD participants, we performed the following control analyses using regressive models.

#### Association between the rsEEG alpha source activity and MMSE in Healthy, PDD, and DLB patients

To underline the clinical relevance of rsEEG alpha rhythms, as a control analysis, a Spearman test (*p* < 0.05) evaluated the correlation between the MMSE score, as an index of the global cognitive status, and the rsEEG alpha 2 and alpha 3 source activity.

Firstly, we performed a correlation analysis considering all Healthy, PDD, and DLB individuals as a whole group for two reasons. On the one hand, the hypothesis was that rsEEG alpha source activity may correlate with the global cognitive status of seniors, including cases with both normal and impaired cognitive functions. On the other hand, the correlation study would have had a low statistical sensitivity if performed only in the separate groups, owing to the limited scatter of global composite cognitive scores within a given group.

**Fig. 5:**
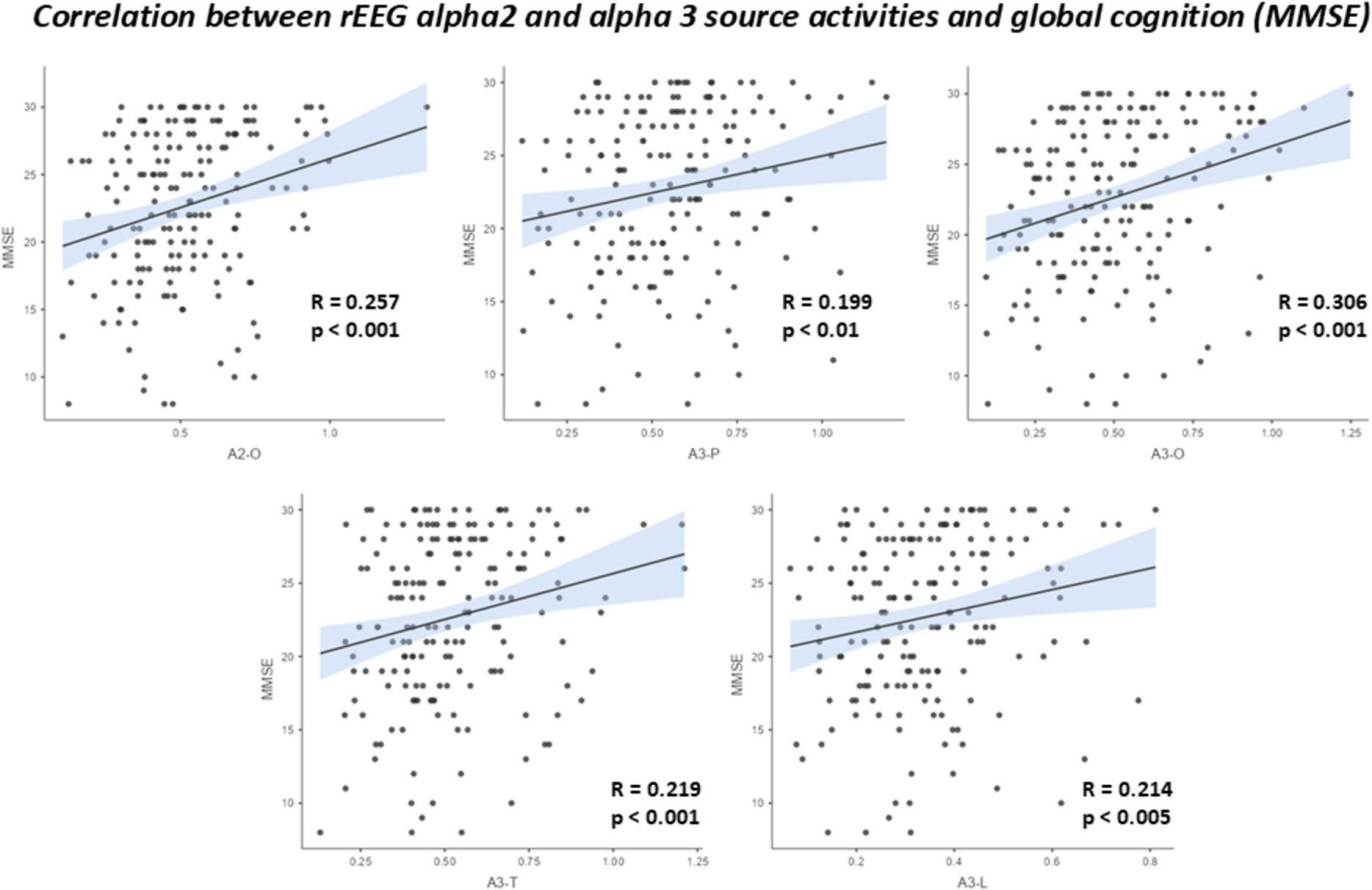
Scatterplot showing the (positive) linear Spearman test correlation between (1) the posterior (parietal, occipital, temporal, and limbic) rsEEG (eLORETA) alpha 2 and alpha 3 source activity and (2) the Mini-Mental State Evaluation (MMSE) score. This correlation was computed in all participants of the present study (*N* = 179)

Figure 5 illustrates the scatterplot showing the statistically significant positive correlation between the MMSE score and the posterior rsEEG (eLORETA) alpha 2 and alpha 3 source activity in all Healthy, DLB, and PDD participants (i.e., Healthy + ADD + PDD) as a whole group. The higher the posterior rsEEG (eLORETA) alpha 2 and alpha 3 source activity, the higher the MMSE score.

Secondly, we evaluated whether any statistically significant difference in the association between the rsEEG alpha source activity and MMSE depending on the Group (Healthy, DLB, and PDD) and Education (Edu-, Edu+) factors may occur. To this aim, several regression models were developed with the following design.

- Target variable: MMSE.
- Predictors: Group (Healthy, DLB, PDD), Education (Edu-, Edu+), posterior rsEEG alpha 2 and alpha 3 source activities, and their interaction.
- Covariates: Age, Sex, Unit.

The model accuracy was assessed by evaluating the residual standard error (RSE) and adjusted *R*-squared (*R*^2^). The residuals’ distribution was checked for heteroscedasticity, normality, and influential observations in the data (*p* < 0.05). The post hoc analysis was performed through the false discovery rate (FDR) correction (*p* < 0.05).

No statistically significant interaction between Group and Education was observed in the association between rsEEG alpha source activity and MMSE (*p* > 0.05).

#### Association between the rsEEG alpha source activity and cognitive functions in PDD patients as a function of educational attainment

To test whether the observed effect in the PDD group may effectively reflect a compensatory effect of the educational attainment (as a proxy of CR), we checked for the association among the educational attainment, the rsEEG alpha 2 and alpha 3 source activities, and the composite cognitive scores computed from the neuropsychological tests, as well as MMSE as a measure of the global cognitive status. To this aim, we computed composite cognitive scores, tested their differences between the control and experimental groups, and performed the mentioned regression analysis.

The composite cognitive scores were calculated as *z*-scores in relation to the values of the Healthy group in the following cognitive domains: language, visuospatial, attention/executive, memory, and global (average among the previous ones) functions. Figure SM8 reports the individual values of the composite cognitive scores, as well as MMSE, for each Edu- and Edu+ subgroup of Healthy, PDD, and DLB participants.

We performed a non-parametric one-way Kruskal-Wallis H test to evaluate the effect of the Group (Healthy, PDD, DLB) factor on each composite cognitive score and MMSE (*p* < 0.05). The multiple comparisons* z*-value test was used for post-hoc analysis (*p* < 0.05). Table 6 illustrates the results of these statistical comparisons among the Healthy, PDD, and DLB groups. As expected, a statistically significant effect of the factor Group was observed in all the composite cognitive scores, with the patients (PDD and DLB) performing worse than Healthy participants (*p* < 0.01). No statistically significant differences were observed between PDD and DLB groups (*p* > 0.05).

**Table 6:** Results of the statistical non-parametric Kruskal–Wallis H tests on the cognitive scores among the Healthy, PDD, and DLB groups. The multiple comparisons *z*-value was used for post hoc analysis (*p* < 0.05). Legend: *Healthy*, cognitively unimpaired older persons; *PDD*, Parkinson’s disease dementia; *DLB*, dementia due to Lewy body disease; *n.s.*, not significant (*p* > 0.05)

Cognitive scores					
			Nold vs. DLB	Nold vs. PDD	DLB vs. PDD
Language	H (2, 176) = 88.3 *p* < 0.01	Nold > DLB, PDD	*p* < 0.01	*p* < 0.01	n.s
Visuospatial functions	H (2, 121) = 43.4 *p* < 0.01	Nold > DLB, PDD	*p* < 0.01	*p* < 0.01	n.s
Attention/executive functions	H (2, 136) = 57.0 *p* < 0.01	Nold > DLB, PDD	*p* < 0.01	*p* < 0.01	n.s
Memory	H (2, 177) = 89.5 *p* < 0.01	Nold > DLB, PDD	*p* < 0.01	*p* < 0.01	n.s
Global composite score	H (2, 177) = 99.6 *p* < 0.01	Nold > DLB, PDD	*p* < 0.01	*p* < 0.01	n.s
MMSE	H (2, 177) = 101.0 *p* < 0.01	Nold > DLB, PDD	*p* < 0.01	*p* < 0.01	n.s

Several linear regression models were performed with the R software (https://www.r-project.org/) with the following design.

- Target variable: the cognitive score (e.g., language, visuospatial, attention/executive, memory, global, and MMSE).
- Predictors: Education (PDD-Edu-, PDD-Edu+), posterior rsEEG alpha 2 and alpha 3 source activities, and their interaction.
- Covariates: Age, Sex, Unit.

The model accuracy was assessed by evaluating the residual standard error (RSE) and adjusted *R*-squared (*R*^2^). The residuals’ distribution was checked for heteroscedasticity, normality, and influential observations in the data (*p* < 0.05). The post-hoc analysis was performed through the false discovery rate (FDR) correction (*p* < 0.05).

Table 7 illustrates the results of the regression analysis. Only for the attention/executive function scores, a statistically significant interaction between the Education predictor and the occipital rsEEG alpha 3 and limbic rsEEG alpha 2 source activities was observed (*p* < 0.05).

**Table 7:** Results of the linear regression model showing the effect on the attention/executive function score (target variable) of the Education, the occipital rsEEG alpha 3 (A3 O) and the limbic rsEEG alpha 2 (A2 L), and their interaction (predictors) in the PDD group. The age, sex, and the clinical unit were used as covariates. Legend: *PDD*, Parkinson’s disease dementia; *rsEEG*, resting state electroencephalographic; *RSE*, residual standard error; *n.s.*, not significant (*p* > 0.05)

Model	Predictors	*β* (± SE)	*p*-value
Attention ~ Edu group * A3 O + Age + Unit + Sex Multiple *R*^2^ = 0.3667 Adjusted *R*^2^ = 0.2841 F-statistic (6, 46) = 4.44 RSE = 4.42 *p*-value = 0.001272	(Intercept)	− 2.47 (± 5.48)	n.s
	Education	9.33 (± 2.90)	*p* < 0.01
	A3 O	10.25 (± 3.83)	*p* < 0.01
	Education * A3 O	− 10.25 (± 4.83)	*p* < 0.05
Attention ~ Edu group * A2 L + Age + Unit + Sex Multiple *R*^2^ = 0.3606 Adjusted *R*^2^ = 0.2772 F-statistic (6, 46) = 4.44 RSE = 4.33 *p*-value = 0.001539	(Intercept)	− 5.99 (± 6.08)	n.s
	Education	13.59 (± 4.62)	*p* < 0.01
	A3 O	23.36 (± 9.09)	*p* < 0.01
	Education * A2 L	− 22.07 (± 10.60)	*p* < 0.05

**Fig. 6:**
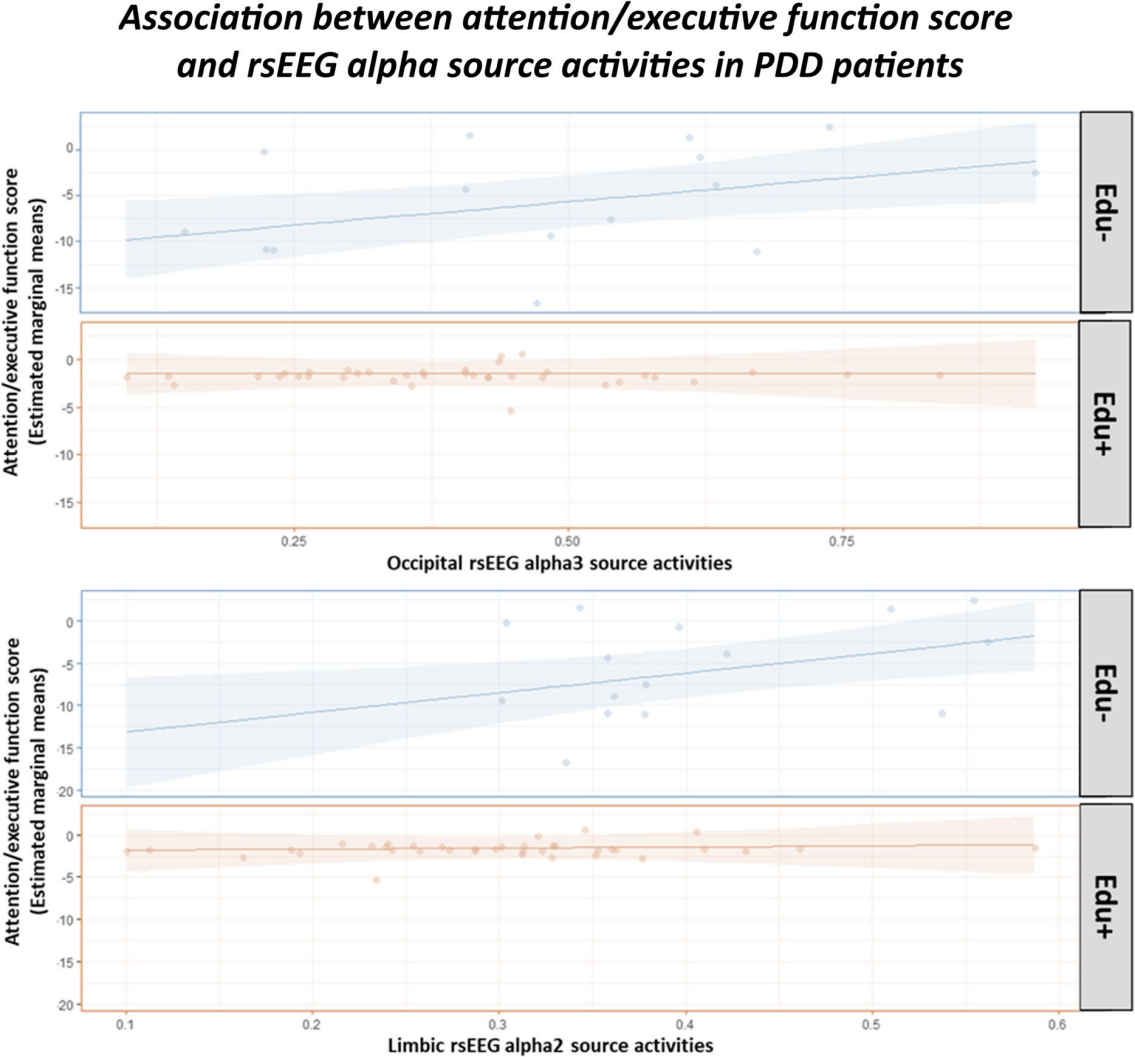
Plot of the association between the attention/executive function score (estimated marginal means) and occipital/limbic rsEEG alpha 3 source activities in the PDD-Edu- and PDD-Edu+ subgroups, as revealed by the linear regression model. Only for the PDD-Edu- subgroup, there were statistically significant positive associations between the attention/executive function score and (upper graph) occipital rsEEG alpha 3 source activities (trend = 10.25 ± 3.83 standard error, SE; lower confidence limit = 2.54; upper confidence limit = 17.95, *p* < 0.01; FDR corrected), and (lower graph) limbic rsEEG alpha 2 source activities (trend = 23.4 ± 9.09 standard error, SE; lower confidence limit = 5.06; upper confidence limit = 41.7, *p* < 0.01; FDR corrected). No statistically significant association was observed for the PDD-Edu+ subgroup (*p* > 0.05). In the model, the age, sex, and the clinical unit were used as covariates. Legend: *PDD*, Parkinson’s disease dementia; *rsEEG*, resting-state electroencephalographic

As depicted in Figure 6, the post hoc analyses revealed that only the PDD-Edu- subgroups showed statistically significant positive associations between the attention/executive function score and (i) occipital rsEEG alpha 3 source activities (trend = 10.25 ± 3.83 standard error, SE; lower confidence limit = 2.54; upper confidence limit = 17.95, *p* < 0.01) and (ii) limbic rsEEG alpha 2 source activities (trend = 23.4 ± 9.09 standard error, SE; lower confidence limit = 5.06; upper confidence limit = 41.7, *p* < 0.01). No statistically significant association was observed for the PDD-Edu+ subgroup (*p* > 0.05).

#### Association between the education attainment and rsEEG alpha source activities in the Healthy, PDD, and DLB participants

Several linear regression models were performed with the R software in each group of interest (i.e., Healthy, PDD, and DLB) with the following design:

- Target variable: global rsEEG alpha 2 source activity, global rsEEG alpha 3 source activity, occipital rsEEG alpha 2 source activity, or occipital rsEEG alpha 3 source activity.
- Predictor: Education attainment (years) as a continuous variable.
- Covariates: Age, sex, and recording unit.

The model accuracy was assessed by evaluating the residual standard error (RSE) and adjusted *R*-squared (*R*^2^). The residuals’ distribution was checked for heteroscedasticity, normality, and influential observations in the data (*p* < 0.05).

The results are reported in the following:

In the Healthy group, there were statistically significant *positive* associations between Education attainment and the following rsEEG variables: occipital alpha 3 (trend = 0.015 ± 0.008 SE; *p* < 0.05), global alpha 2 (trend = 0.010 ± 0.005 SE; *p* < 0.05), and alpha 3 source activities (trend = 0.015 ± 0.005 SE; *p* < 0.01), in line with the possible effect of neurophysiological neuroprotective mechanisms.

In the PDD group, there were statistically significant *negative* associations between Education attainment and the following rsEEG variables: occipital alpha 2 (trend = −0.015 ± 0.005 SE; *p* < 0.01), occipital alpha 3 (trend = −0.014 ± 0.003 SE; *p* < 0.05), and global alpha 2 (trend = −0.014 ± 0.003 SE; *p* < 0.01) source activities, in line with the possible effect of compensatory mechanisms.

No statistically significant effect was found in the DLB participants (*p* > 0.05).

### Cross-validation on other cohorts of Healthy, PD with cognitive deficits, and LB with cognitive deficits

In the Supplementary Materials Results, we reported the demographical and clinical information about the Healthy (*n* = 54; Table SM1), PD with cognitive deficits (mild cognitive impairment, MCI, and dementia, PDCD; *N* = 38; Table SM2), and LB with cognitive deficits (LBCD; *N* = 44; Table SM3) participants from an independent database for cross-validation purposes. In that database, inclusion/exclusion criteria and the clinical-neuropsychological protocol were quite similar to those of the main experiment. Furthermore, the rsEEG recordings were performed with 19 scalp electrodes placed according to a 10–20 montage system and the same recording protocol. The same rsEEG analysis was performed.

Briefly, similar patterns of the statistical differences between low (Edu-) and high (Edu+) educational attainment subgroups observed in the main analyses were found. In Healthy participants, there was a statistically significant ANOVA interaction effect (*F*(7, 343) = 3.1610, *p* < 0.01) between the factors Education (Healthy-Edu- and Healthy-Edu+; dependent variable) and Band (delta, theta, alpha 1, alpha 2, alpha 3, beta 1, beta 2, and gamma). The Duncan planned post-hoc (*p* < 0.05 Bonferroni correction for 7 frequency bands, *p* < 0.05/7 = 0.007) test showed that the discriminant pattern Healthy-Edu+ > Healthy-Edu- was fitted by the rsEEG alpha 2 and alpha 3 (*p* < 0.05 Bonferroni corrected) source activities. No other effects involving the factor Education were observed (*p* > 0.05; Figure SM8).

Along the same line, in the PDCD group, a statistically significant ANOVA interaction effect (*F*(7, 490) = 3.05, *p* < 0.01) between the factors Education (PDCD-Edu- and PDCD-Edu+; dependent variable) and Band (delta, theta, alpha 1, alpha 2, alpha 3, beta 1, beta 2, and gamma). The Duncan planned post hoc (*p* < 0.05 Bonferroni correction for 7 frequency bands, *p* < 0.05/7 = 0.007) testing showed that the discriminant pattern PDCD-Edu- > PDCD-Edu+ was fitted by the rsEEG alpha 2 source activity (*p* < 0.05 Bonferroni corrected). No other effects involving the factor Education were observed (*p* > 0.05; Figure SM9).

Finally, no statistically significant two- and three-way ANOVA interaction effects were observed in the DLCD group (*p* > 0.05).

### Control analysis on the correlation between motor impairment and rsEEG source activities

To evaluate whether the compensatory mechanism revealed by rsEEG rhythms in the PDD group may depend on motor dysfunctions, we explored the correlation between motor impairment (UPDRS III) and rsEEG theta and alpha source activities. The theta band was included as associated with greater motor impairment in PD patients when recorded from sensorimotor cortical areas [91].

To this aim, we developed several Pearson’s correlation models between the UPDRS III score and the regional (frontal, central, parietal, occipital, and temporal) and global rsEEG theta, alpha 2, and alpha 3 source activities estimated in the PDD and DLB groups. To include as many patients as possible, we pulled together the experimental and the cross-validation cohort. For the sake of simplicity, we will use the abbreviations PDD and DLB even if the cross-validation cohort was composed of patients with MCI and dementia. The results of these analyses are reported in the Supplementary Materials. Only for the extended PDD group (*N* = 104) statistically significant positive correlations were observed between the regional and global rsEEG theta source activities and the UPDRS III score; Pearson’s *r* ranged between 0.19 and 0.30 (*p* < 0.05; Figure SM11). No statistically significant correlations were observed for the extended DLB group (*N* = 83; *p* > 0.05; Figure SM12). Concerning rsEEG alpha 2 and alpha 3 source activities, no statistically significant correlations with the UPDRS III score were observed for the extended PDD (Table SM5) and DLB (Table SM6) groups.

## Discussion

In this study, we explored the relationship between educational attainment, a CR proxy, and rsEEG source activity in patients with PDD and DLB. Our goal was to evaluate the potential neuroprotective, still acting even after the onset of cognitive deficits, and compensatory (resilient) mechanisms that could influence this activity, which is crucial for the regulation of cortical arousal and vigilance during quiet wakefulness [39].

Our findings in cognitively unimpaired older adults, who served as a control group, showed that those with higher educational attainment exhibited significantly greater rsEEG alpha source activity compared to those with lower educational attainment. These results are consistent with previous studies by our Consortium using similar methodologies [39–41] and to role played by the early-life education in protecting brain health and preventing the onset of age-related cognitive decline. This increased rsEEG alpha activity may reflect CR-dependent neuroprotective mechanisms that enhance the brain’s alpha-frequency neuronal oscillations, potentially supporting cortical arousal and vigilance regulation.

A novel aspect of our study is the observation that PDD patients with higher educational attainment demonstrated lower rsEEG alpha source activity, indicative of vigilance dysregulation. Yet, they maintained a similar cognitive status to PDD patients with lower educational attainment. This effect could not be attributed to age, sex, or overall cognitive-motor status, suggesting the involvement of CR-dependent compensatory (resilient) mechanisms for cognitive status against a greater derangement of neural systems generating rsEEG alpha rhythms in patients with higher educational attainment, as previously observed in Alzheimer’s disease (AD) patients [39–41]. This hypothesis is further supported by the positive correlation between rsEEG alpha source activity and attention/executive function scores in PDD patients with lower educational attainment, a relationship not observed in those with higher educational attainment, possibly due to CR’s mediating role. However, the complex interplay of CR with genetic, neuropathological, neurodegenerative, sociocultural, environmental, and other factors remains unclear at the present early stage of the research.

### A speculative model explaining more abnormal rsEEG alpha rhythms in PDD patients with higher educational attainment

Nearly a century after Hans Berger discovered human rsEEG alpha rhythms [92], the following previous EEG and fMRI studies suggest that the neuroprotective and compensatory mechanisms inferred from our rsEEG findings in healthy controls and PDD patients may involve reciprocal thalamic-cortical circuits, subcortical ascending systems, and cortical neural networks responsible for generating EEG activity. Specifically, previous EEG and fMRI findings in healthy individuals indicated that dominant rsEEG alpha rhythms might reflect tonic inhibition in posterior cortical regions, primarily mediated by cortico-cortical and reciprocal cortico-thalamic circuits [39, 93–97]. Additionally, these studies found that highly educated individuals exhibited enhanced functional coupling of widespread rsEEG alpha rhythms [98, 99] and stronger associations between posterior rsEEG alpha rhythms and rs-fMRI connectivity along thalamic and visual circuits [41]. Thus, it is plausible that higher educational attainment in healthy older persons is associated with neurophysiological neuroprotective mechanisms that enhance these circuits, supporting cortical arousal and vigilance regulation.

In contrast, our findings of reduced rsEEG alpha rhythms in PDD patients with higher educational attainment may be due to a disruption of critical subcortical and cortical circuits with some compensatory (resilient) mechanisms able to compensate for it and maintain cognitive status in relation to PDD patients with lower education attainment. Previous EEG and neuroimaging studies have attributed the slowing of rsEEG alpha rhythms and the dominance of theta and delta rhythms (< 6 Hz) in patients with AD, PDD, and DLB to diminished inputs from cholinergic, noradrenergic, and dopaminergic arousal systems [39, 66–75]. For instance, posterior rsEEG alpha rhythms in AD patients were modulated by both acute [100] and chronic [101] administration of acetylcholinesterase inhibitors and noradrenergic deficits in the thalamus [102]. In PD patients with cognitive decline, rsEEG alpha rhythms and motor performance were influenced by levodopa treatment [103–105]. Furthermore, these patients exhibited a negative correlation between occipital rsEEG alpha frequency and [11 C]yohimbine PET, reflecting α2 adrenoceptor density in the thalamus [106].

Previous studies also suggest that PDD patients with higher educational attainment may compensate (be resilient) for the disruption of cholinergic, noradrenergic, and dopaminergic systems through several structural and functional neuroprotective mechanisms that partially preserve cognitive status. These mechanisms include relatively preserved MRI-based gray matter volume in the basal ganglia and dorsal prefrontal cortex [62], FDG-PET metabolism in the right fusiform gyrus and other cortical areas [57], dopamine transporter binding in the basal ganglia [63], and rs-fMRI functional connectivity in the anterior cingulate, basal ganglia, and frontoparietal regions [64]. Additionally, studies in PD patients with cognitive deficits have shown associations between impaired executive function, rsEEG slowing, and reduced [18 F]FDOPA PET uptake, as well as reduced gray matter volume in the cholinergic basal forebrain nuclei [107, 108].

Another interesting aspect worth discussing is that the disease duration was shorter in the PDD patients with higher than lower educational attainment. Indeed, the PDD-Edu+ subgroup was characterized by a delayed disease onset, followed by a more accelerated cognitive and functional decline. This is in line with previous evidence that higher education delays the onset of degenerative diseases in aging, possibly due to neuroprotective processes of cognitive reserve [108]. However, this delay is not necessarily related to a lower neuropathological burden (i.e., brain amyloidosis), so the cognitive decline may be faster when the cognitive reserve is exhausted [38, 57].

Unfortunately, we lack structural and functional neuroimaging data to fully understand why the rsEEG activity in DLB patients did not differ with educational attainment levels. We can only speculate that in the present cohorts, the DLB patients with higher educational attainment may have exhausted the CR-related compensatory mechanisms due to heavier neuropathological and neurodegenerative burdens with eventual implications on cholinergic systems than those possibly affecting PDD patients. In this speculative line, previous neuroimaging studies in DLB patients have shown significant negative associations between CR indices and FDG-PET metabolism in key brain regions [58–60], as well as between CR indices and functional connectivity in cortical networks associated with core clinical features of DLB [52, 61]. Furthermore, they often reported heavy co-pathologies in DLB compared to PDD patients, such as AD-related neurofibrillary tangles and Aβ plaques [109–112] or frontotemporal lobar degeneration (FTLD [113]). Moreover, DLB patients were found to be characterized by more severe neuronal loss and the predominant involvement of the medial portion of the substantia nigra (which projects to the caudate nucleus) as compared to PDD patients [114]. Finally, meta-analyses revealed more beneficial clinical effects of cholinesterase inhibitors in PDD patients, possibly reflecting more responsivity of residual cholinergic neurons than in DLB patients [115, 116]. The disruptive effect of the burden of these co-pathologies may also lead to more rapid cognitive decline over time [112], possibly reducing the time for CR to induce neuroplasticity and reorganization of brain functional connectivity to maintain cognitive status. This would be consistent with the shorter disease duration of the DLB compared to the PDD observed in the present study cohort, suggesting a more aggressive and rapid neurodegenerative phenomenon. Future longitudinal data collection within these patients is required to highlight the eventual differential relation between the underlying neurobiological pathology, neuronal loss, and the effect of educational attainment or other more comprehensive CR indexes.

### Methodological considerations

This retrospective and exploratory study utilized clinical and rsEEG datasets from the Consortium’s archive (www.pdwaves.eu). It is based on data from multiple clinical units that did not follow harmonized data collection procedures as would be done in a prospective clinical trial. Furthermore, the clinical units only belonged to the Western Eurasia area, which limits the generalizability of our results to the world population. Based on the limited composition of the available cohort, we could only perform a control analysis using a linguistic-cultural background factor with two levels, namely Europe and Turkey. Results showed no effect of the linguistic-cultural background factor in the present design. However, we know that the relationship between educational attainment and cognitive reserve may be moderated by socioeconomic factors, as well as lifelong cultural and occupational activities. All of these aspects may vary considerably across countries, and an extension of the sample, including participants from different socio-economic and linguistic-cultural backgrounds, is required for future cross-validation.

Furthermore, we had access to a relatively small number of PDD and DLB datasets. Given the exploratory nature of this study, we employed a liberal statistical threshold of *p* < 0.05 in some analyses. The relatively small sample size did not allow us to directly compare the PDD and DLB groups due to their well-known different demographic features (e.g., age and sex). Furthermore, the limited number of Healthy, DLB, and PDD participants did not allow us to use the same thresholds of low and high educational attainment in the Healthy, DLB, and PDD groups of the main statistical analysis. This problem was partially mitigated by the fact that those thresholds were very similar in the DLB and PDD groups. Indeed, both pathological groups showed mean low and high educational attainment levels of about 5 and 10 years, respectively. The mean of 5 years corresponds to the first cycle of school in most European countries, while the mean of 10 years includes subsequent cycles. However, we performed a control analysis that matched the educational attainment levels of the Edu- and Edu+ subgroups across the Healthy, DLB, and PDD participants. Results confirmed those obtained on the whole cohort.

In this study, educational attainment was used as a proxy for CR due to its simplicity, availability, and consistency in neurodegenerative disease research [15, 16]. Future studies should consider a broader range of CR indices, including those based on lifetime occupation and leisure activities, as well as residual cognitive performance after accounting for neuropathological and neurodegenerative factors [55].

In the present PDD group, residual cholinergic activity may contribute to the compensatory mechanisms maintaining cognitive status in patients with higher education attainment against more deranged rsEEG alpha rhythms when compared to patients with lower education attainment. Unfortunately, we could not test this possible explanation with molecular, structural, or functional neuroimaging evidence, making speculative the interpretation of the results.

The rsEEG recordings in this study were obtained using a 10–10 electrode montage system with 30 scalp electrodes. This may be sufficient for exploratory studies using source estimation techniques at low spatial resolution [39]. However, this approach limits the precision of cortical source localization, which was performed across large cortical regions of interest (e.g., cortical lobes) using eLORETA. While this method is suitable for modeling spatially widespread cortical source activations, future studies should employ high-resolution EEG techniques with 64–256 scalp electrodes to achieve higher spatial resolution in rsEEG source estimation [117, 118].

Finally, the rsEEG recordings were conducted in a single session, precluding longitudinal evaluation of CR effects on rsEEG rhythms in PDD and DLB patients. Future studies should adopt longitudinal, multicentric, and prospective designs, incorporating large cohorts of PDD and DLB patients and more conservative statistical thresholds to reduce the risk of false-positive findings. Additionally, multimodal approaches combining rsEEG, MRI, and FDG-PET with harmonized longitudinal data collection procedures and biomarkers will likely offer a more comprehensive understanding of CR effects over the disease course.

## Conclusions

This study investigated whether educational attainment, a typical CR proxy, influences rsEEG rhythms reflecting neurophysiological mechanisms underlying cortical arousal and vigilance regulation in PDD and DLB patients. For this purpose, cognitively unimpaired older adults served as a control group. Participants in each group were divided into low (Edu-) and high (Edu+) educational attainment subgroups.

In the Healthy group, participants with higher educational attainment (Edu+) exhibited significantly greater rsEEG alpha rhythms compared to those with lower educational attainment (Edu), potentially indicating neuroprotective neurophysiological mechanisms. In contrast, within the PDD group, widespread rsEEG alpha rhythms were lower in Edu+ patients compared to Edu- patients. This reduction in the rsEEG alpha activity might reflect compensatory mechanisms that help maintain cognitive-motor status in Edu+ patients despite the underlying disruption of such a rsEEG activity. Meanwhile, in the DLB group, no significant differences in rsEEG rhythms were observed between Edu+ and Edu- patients.

Our results suggest that in PDD patients, but not in DLB patients, higher educational attainment may compensate for abnormalities in the brain’s neurophysiological oscillatory mechanisms that underlie rsEEG alpha rhythms and the regulation of vigilance. These findings underscore the profound significance of early-life education as a crucial component in strategies to fortify brain reserve against neurodegenerative diseases. This compelling evidence highlights how investing in education from a young age plays a pivotal role in bolstering cognitive resilience and protecting against the onset of these debilitating neurodegenerative conditions. More specifically, early-life education may represent one of the best investments of national governments, especially in lower-income countries, for the prevention of cognitive deficits in PDD patients along aging, mitigating the unbearable social and economic burden of that pathological condition.

Future studies that combine rsEEG with neuroimaging techniques should explore the metabolic, neuromodulatory (e.g., cholinergic), and functional connectivity correlates of these hypothesized compensatory (resilient) mechanisms in the PDD brain. Additionally, research should investigate whether the absence of these compensatory effects in DLB patients is due to more severe dysfunctions in the associated neural correlates.

## Supplementary Information

Below is the link to the electronic supplementary material.

Supplementary file1 (DOCX 1.20 MB)

## Data Availability

The data that support the findings of this study are available on request from the corresponding author. The data are not publicly available due to privacy or ethical restrictions.
