# Supplementary material for: The compensatory effect of education as revealed by resting-state electroencephalographic alpha rhythms in patients with dementia due to Parkinson’s disease: findings from an exploratory study
Source: GeroScience. 2025 Jun 11;48(1):1189–218. doi: 10.1007/s11357-025-01703-9 (PMC12972170; doi:10.1007/s11357-025-01703-9)
Supplement: Supplementary file 1 — Supplementary file1 (DOCX 1.20 MB) [file 11357_2025_1703_MOESM1_ESM.docx]

***Supplementary Materials***

**The compensatory effect of education as revealed by resting-state electroencephalographic alpha rhythms in patients with dementia due to Parkinson's disease: findings from an exploratory study**

Susanna Lopez^1^, Claudio Del Percio^1^, Roberta Lizio^1^, Giuseppe Noce^2^, Dharmendra Jakhar^1^, Andrea Soricelli^2,3^, Marco Salvatore^2^, Bahar Güntekin^4^, Görsev Yener^5^, Federico Massa^6,7^, Dario Arnaldi^6,8^, Francesco Famà^6,8^, Matteo Pardini^6^, Raffaele Ferri^9^, Filippo Carducci^1,9^, Bartolo Lanuzza^9^, Fabrizio Stocchi^10,11^, Laura Vacca^10^, Chiara Coletti^10^, Moira Marizzoni^12^, John Paul Taylor^13^, Lutfu Hanoğlu^14^, Nesrin Helvacı Yılmaz^15^, İlayda Kıyı^16^, Yağmur Özbek-İşbitiren^16^, Anita D’Anselmo^17^, Laura Bonanni^17^, Roberta Biundo^18,19^, Fabrizia D’Antonio^20^, Giuseppe Bruno^20^, Angelo Antonini^19^, Franco Giubilei^22^, Sofia Cuoco^23^, Paolo Barone^23^, Giovanni B. Frisoni^24,25^, Rossella Rotondo^26^, Francesca De Pandis^11,26^, and Claudio Babiloni^1,26*^

*^1^ Department of Physiology and Pharmacology “Vittorio Erspamer,” Sapienza University of Rome, Rome, Italy;*

*^2^ IRCCS Synlab SDN, Naples, Italy;*

*^3^ Department of Medical, Movement and Well-being Sciences, University of Naples Parthenope, Naples, Italy;*

*^4^ Department of Biophysics, School of Medicine, Istanbul Medipol University, Istanbul, Turkey;*

*^5^ Izmir University of Economics, Faculty of Medicine, Izmir, Turkey;*

*^6^ Dipartimento di Neuroscienze, Oftalmologia, Genetica, Riabilitazione e Scienze Materno-infantili (DiNOGMI), Università di Genova, Italy;*

*^7^ Clinica neurologica, IRCCS Ospedale Policlinico San Martino, Genova, Italy;*

*^8^ Neurofisiopatologia, IRCCS Ospedale Policlinico San Martino, Genova, Italy*

*^9^ Oasi Research Institute – IRCCS, Troina, Italy;*

*^10^* *IRCCS San Raffaele, Rome, Italy;*

*^11^ Telematic University San Raffaele Rome, Rome, Italy.*

*^12^* *Biological Psychiatry Unit, IRCCS Istituto Centro San Giovanni di Dio Fatebenefratelli, Brescia, Italy;*

*^13^ Translational and Clinical Research Institute, Faculty of Medical Sciences, Newcastle University, UK;*

*^14^ Department of Neurology, School of Medicine, Istanbul Medipol University, Istanbul, Turkey;*

*^15^ Parkinson’s Disease and Movement Disorders Center (PARMER), Istanbul Medipol University, Istanbul, Turkey;*

*^16^ Health Sciences Institute, Department of Neurosciences, Dokuz Eylül University, Izmir, Turkey;*

*^17^ Department of Aging Medicine and Sciences, University “G. d’Annunzio” of Chieti-Pescara, Italy;*

*^18^ Parkinson and Movement Disorders Unit, Study Center for Neurodegeneration (CESNE), Center for Rare Neurological*

*Diseases (ERN RND), Padua, Italy; Department of Neuroscience, University of Padua, Padua, Italy;*

*^19^ Department of Neuroscience, University of Padua, Padua, Italy;*

*^20^* *Department of General Psychology, University of Padua, Padova, Italy;*

*^21^ Department of Human Neurosciences, Sapienza University of Rome, Rome, Italy;*

*^22^Department of Neuroscience, Mental Health, and Sensory Organs, Sapienza University of Rome, Rome, Italy;*

*^23^ Department of Medicine, Surgery and Dentistry “Scuola Medica Salernitana”, Neuroscience Section, University of Salerno, Baronissi, Italy;*

*^24^ Laboratory of Neuroimaging of Aging (LANVIE), University of Geneva, Geneva, Switzerland;*

*^25^ Geneva Memory Center, Department of Rehabilitation and Geriatrics, Geneva University Hospitals, Geneva, Switzerland;*

*^26^ Hospital San Raffaele Cassino, Cassino (FR), Italy;*

**Corresponding author:** Prof. Claudio Babiloni

Department of Physiology and Pharmacology "V. Erspamer"

Sapienza University of Rome

P. le A. Moro 5, 00185, Rome, Italy

Phone: +39 0649910989

E-mail: [claudio.babiloni@uniroma1.it](mailto:claudio.babiloni@uniroma1.it)

**Running title:** rsEEG depends on education in Parkinson’s Disease Dementia.

**Keywords**: Parkinson’s Disease Dementia (PDD); Lewy Body Dementia (DLB), Resting-State Electroencephalographic (EEG) Rhythms; Alpha Rhythms; Exact Low-Resolution Brain Electromagnetic Source Tomography (eLORETA); Education; Cognitive reserve.

**Supplementary Materials and Methods**

*Cross-validation cohort participants*

The clinical and resting-state electroencephalographic (rsEEG) datasets used for cross-validation were sourced from the Eurasian archive of the PDWAVES Consortium ([www.pdwaves.eu](http://www.pdwaves.eu)) and the European Dementia with Ley Bodies (DLB) Consortium. These datasets included records from 54 Healthy participants, 38 Parkinson’s disease (PD) participants, and 44 participants with DLB, all of whom had undergone rsEEG recordings under eyes-closed conditions. To ensure a sufficient sample size, we included patients at both the prodromal (mild cognitive impairment, MCI) and dementia stages for PD and DLB, collectively referred to as “MCI+D.” Specifically, these patients were recruited from the following clinical units of the PDWAVES Consortium: Sapienza University of Rome (Italy), University “G. d’Annunzio” of Chieti-Pescara (Italy), Institute for Research and Evidence-based Care (IRCCS) “Fatebenefratelli” of Brescia (Italy), IRCCS Synlab SDN of Naples (Italy), Oasi Research Institute-IRCCS in Troina (Italy), IRCCS Ospedale Policlinico San Martino and DINOGMI (University of Genova, Italy), Hospital San Raffaele of Cassino (Italy), IRCCS San Raffaele Pisana of Rome (Italy), Translational and Clinical Research Institute of Newcastle University (UK), Izmir University of Economics, Faculty of Medicine (Turkey), and Medipol University of Istanbul (Turkey).

To assess the impact of educational attainment as a proxy for cognitive reserve (CR) on rsEEG source activities, the enrolled Healthy, PD(MCI+D), and DLB(MCI+D) participants were stratified into sub-groups based on the median educational attainment of each group: Healthy-Edu- (N = 27; <10 years), Healthy-Edu+ (N = 27; ≥10 years), PD(MCI+D)-Edu- (N = 19; <10 years), PD(MCI+D)-Edu+ (N = 19; ≥10 years), DLB(MCI+D)-Edu- (N = 22; <10 years), and DLB(MCI+D)-Edu+ (N = 22; ≥10 years). Within each group (i.e., Healthy, PD(MCI+D), and DLB(MCI+D)), the Edu- and Edu+ subgroups were matched for age, sex, and global cognitive status, ensuring that the subgroups had identical mean values for age, education, and MMSE score.

The study was approved by the local institutional ethical committees. All experiments were conducted in accordance with the Code of Ethics of the World Medical Association (Declaration of Helsinki) and the standards established by the local institutional review boards. Informed and explicit consent was obtained from each participant or their caregiver prior to participation.

*Diagnostic criteria*

The diagnosis of Parkinson’s disease (PD) (N = 38) was based on a standard clinical assessment of tremor, rigidity, and bradykinesia [1]. The severity of motor disability was measured using the Hoehn and Yahr stage [2] and the Unified Parkinson Disease Rating Scale-III (UPDRS III;[3]) for extrapyramidal symptoms. Unfortunately, data from the Movement Disorder Society-Sponsored Revision of the Unified Parkinson's Disease Rating Scale were not available. Patients with Parkinson's disease dementia (PDD) were identified by a history of dementia preceded by a typical levodopa-responsive Parkinsonian motor syndrome for at least 12 months, unrelated to any other pathological conditions than PD.

The diagnosis of Mild Cognitive Impairment due to Parkinson’s Disease (PDMCI) followed the Diagnostic Criteria for Mild Cognitive Impairment in Parkinson’s Disease [4]. Inclusion criteria included: (1) a confirmed diagnosis of PD as described above; (2) a gradual cognitive decline reported by the patient, an informant, or observed by clinicians in the context of established PD; and (3) cognitive deficits that did not significantly interfere with functional independence in daily activities, although slight difficulties with complex tasks may have been present. Exclusion criteria for PDMCI, based on clinical features and neuroradiological findings, included other forms of parkinsonism: (1) Dementia with Lewy Body (DLB) [5, 6, 7], (2) drug-induced parkinsonism, (3) cerebrovascular parkinsonism, (4) atypical parkinsonism with minimal or no response to antiparkinsonian drugs, and (5) mixed neurodegenerative diseases. All PDMCI subjects underwent a battery of clinical scales and neuropsychological tests, as detailed in [8].

Selected PD patients did not suffer from severe tremors or dyskinesias. The PD diagnosis was made according to the Diagnostic and Statistical Manual of Mental Disorders criteria, fourth edition (DSM-IV-TR; American Psychiatric Association). Inclusion criteria for PDD patients included: (1) a confirmed diagnosis of PD as specified earlier; (2) a gradual neurocognitive decline observed by the patient, a reliable informant, or clinicians in the context of established PD; (3) an abnormally low score in at least one neuropsychological test (defined as performance more than 1.5 standard deviations below the mean for age- and education-matched controls); and (4) moderate to severe impairment in instrumental activities of daily living and dependency. All PDD patients were under standard long-term chronic dopaminergic treatment, and all exams were performed in the ON state.

The diagnosis of DLB (N = 44) was based on consensus guidelines [9]. Clinical features of DLB were assessed as follows: (1) the frequency and severity of hallucinations were evaluated using item 2 of the Neuropsychiatric Inventory (NPI; [10]); (2) the severity of frontal dysfunction was assessed using the Frontal Assessment Battery (FAB; [11]); (3) the presence and severity of cognitive fluctuations were determined using the Clinician Assessment of Fluctuations [12, 13]; (4) extrapyramidal signs were evaluated using the UPDRS-III [3]; and (5) the presence of REM sleep behavior disorder (RBD) was determined based on the minimal International Classification of Sleep Disorders criteria (1992).

The diagnosis of Mild Cognitive Impairment due to DLB (DLBMCI) followed international consensus guidelines for probable DLB at the MCI stage [9, 14]. All but one DLBMCI patient underwent a DAT scan to confirm the DLB diagnosis. Evaluations included the NPI for hallucinations (Cummings et al., 1994), the Clinician Assessment of Fluctuations and FAB for cognitive fluctuations and frontal dysfunction, respectively [11, 12, 13], the UPDRS-III for extrapyramidal symptoms [3], and assessment of RBD based on the International Classification of Sleep Disorders criteria (1992). Inclusion criteria for DLBMCI patients were: (1) age 55–90 years; (2) a gradual cognitive decline observed by the patient, an informant, or clinicians in the context of established DLB; (3) cognitive deficits that did not significantly interfere with functional independence, although slight difficulties with complex tasks may have been present; (4) MMSE score of 24 or higher; (5) Clinical Dementia Rating score of 0.5; and (6) Geriatric Depression Scale (GDS) score of 5 or lower. Exclusion criteria based on clinical and neuroradiological features included: (1) PD [1]; (2) secondary parkinsonism, including drug-induced parkinsonism; (3) cerebrovascular parkinsonism; (4) atypical parkinsonism with minimal or no response to antiparkinsonian drugs; and (5) mixed dementia. Due to the retrospective nature of the study and the varied protocols across clinical units, DLBMCI patients underwent different clinical scales, including the NPI [10], scales for assessing behavioral and psychological symptoms of dementia, MMSE, Dementia Rating Scale-2 [15], Epworth Sleepiness Scale, and the Alzheimer's Disease Cooperative Study Activities of Daily Living (ADCS-ADL). Additionally, DLBMCI patients were evaluated with different neuropsychological tests to assess MCI status [16], covering general cognitive performance in memory, language, executive function/attention, and visuoconstruction abilities (with some receiving the CERAD-plus battery).

Exclusion criteria for healthy seniors included: (1) any neurological or psychiatric disease (past or present), (2) a depressive episode indicated by a Geriatric Depression Scale (15-item version) score higher than 5, (3) use of chronic psychoactive drugs, and (4) significant chronic systemic illnesses such as diabetes mellitus.

The cognitive performance of participants in various domains—including language, visuospatial function, executive function/attention, and memory—was assessed using local neuropsychological test batteries. Specifically: (1) language was tested using the Verbal Fluency Test for letters [17] and the Verbal Fluency Test for categories (e.g., fruits, animals, or car trades; [17]); (2) visuospatial functions were assessed using the Line Orientation Test (Benton et al., 1978) and the Face Recognition Test [18]; (3) executive functions and attention were evaluated using the Trail Making Test Parts A and B [19], the Stroop Test [20], and the Confusion Assessment Method (executive function part; [21]); and (4) memory was tested using the Digit Span Forward and Backward [22], Oktem Verbal Memory Test [23], and the Confusion Assessment Method (memory part; [21]). Each clinical unit administered one or more of the neuropsychological tests for each cognitive domain according to their local protocols.

*The rsEEG recordings*

The rsEEG recordings were conducted using local routine professional digital EEG systems licensed for clinical applications. The specific equipment brands were included as covariates in the statistical models used in this study. All rsEEG recordings were performed in the morning to minimize potential variations due to circadian rhythms. Standard instructions for the resting-state condition emphasized staying awake, remaining psychophysically relaxed with mind wandering and adhering to the experimenter’s requests to keep the eyes closed and open during the rsEEG recording. The experimenters closely monitored the participants' behavioral state during the EEG recordings, noting any deviations or alarms.

EEG data were recorded across all clinical units using a common electrode montage of 19 scalp electrodes, placed according to the 10–20 system, which was also used for data analysis. These electrodes, referred to as "selected electrodes," were positioned to cover the entire scalp (Figure 1). The reference electrode was typically placed between Fz and Cz of the 10-20 system, with the ground electrode positioned on the posterior midline. To minimize the influence of different reference electrode placements, all EEG data were re-referenced to the common average for data analysis.

Electrooculographic (EOG) activity was recorded using a standard bipolar montage to monitor and control eye movements and blinking. As a minimum standard across all clinical units, electrophysiological data allowed for bandpass filtering between 0.3-70 Hz and were sampled at a rate of 256 Hz.

**Supplementary Results**

## Individual distribution of rsEEG source activities in the Edu- and Edu+ subgroups of Healthy, PDD, and DLB participants

The findings mentioned in the main ms were not due to outliers from individual normalized eLORETA current densities (log 10 transformed), as shown by Grubbs' test with an arbitrary threshold of p > 0.001. Supplementary Materials Figure SM1 illustrates the distribution of the individual values for the Healthy-Edu- and Healthy-Edu+ subgroups.

***Figure SM1***


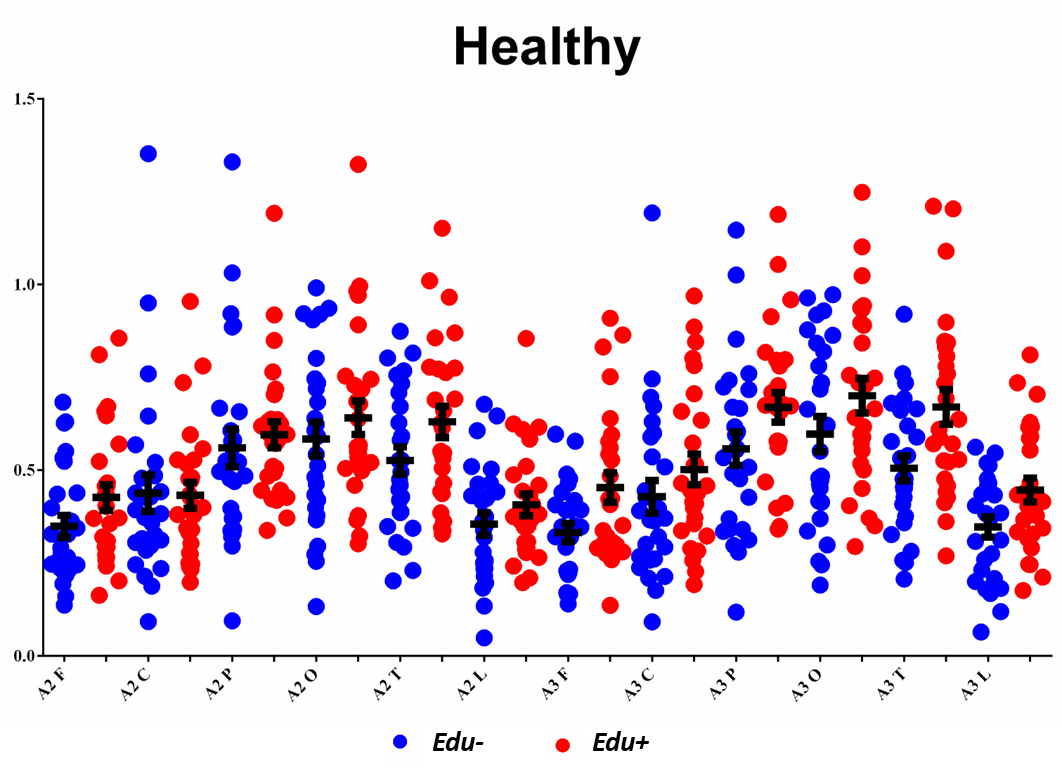


***Figure Supplementary Materials 1 (SM1).*** *Distribution of the individual values of the rsEEG alpha 2 and alpha 3 source activities (normalized eLORETA current density) in the Healthy-Edu- and Healthy-Edu+ subgroups. No outliers were detected according to Grubbs’ test with an arbitrary threshold of p > 0.001. Legend: Healthy = cognitively unimpaired older persons; rsEEG = resting state electroencephalographic.*

Figure SM2 illustrates the distribution of the individual values for the PDD-Edu- and PDD-Edu+ subgroups.

***Figure SM2***

***
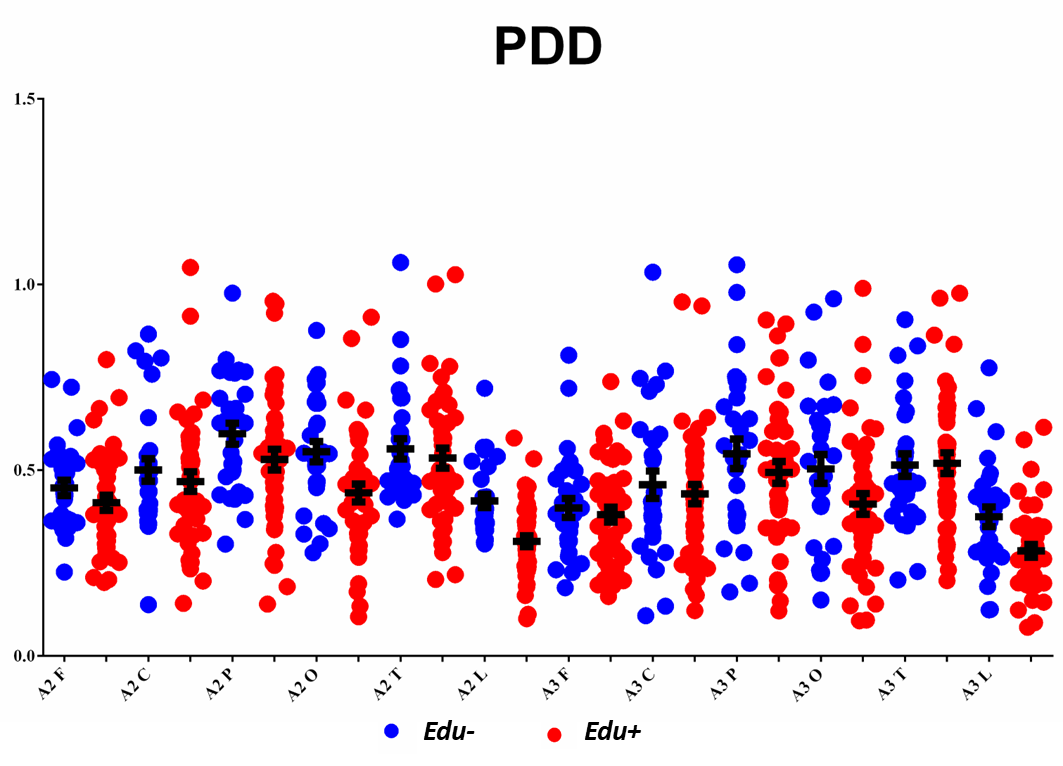
***

***Figure SM2.*** *Distribution of the individual values of the rsEEG alpha 2 and alpha 3 source activities (normalized eLORETA current density) in the PDD-Edu- and PDD-Edu+ subgroups. No outliers were detected according to Grubbs’ test with an arbitrary threshold of p > 0.001. Legend: PDD = Parkinson’s Disease Dementia; rsEEG = resting state electroencephalographic.*

Figure SM3 illustrates the distribution of the individual values for the DLB-Edu- and DLB-Edu+ subgroups.

***Figure SM3***


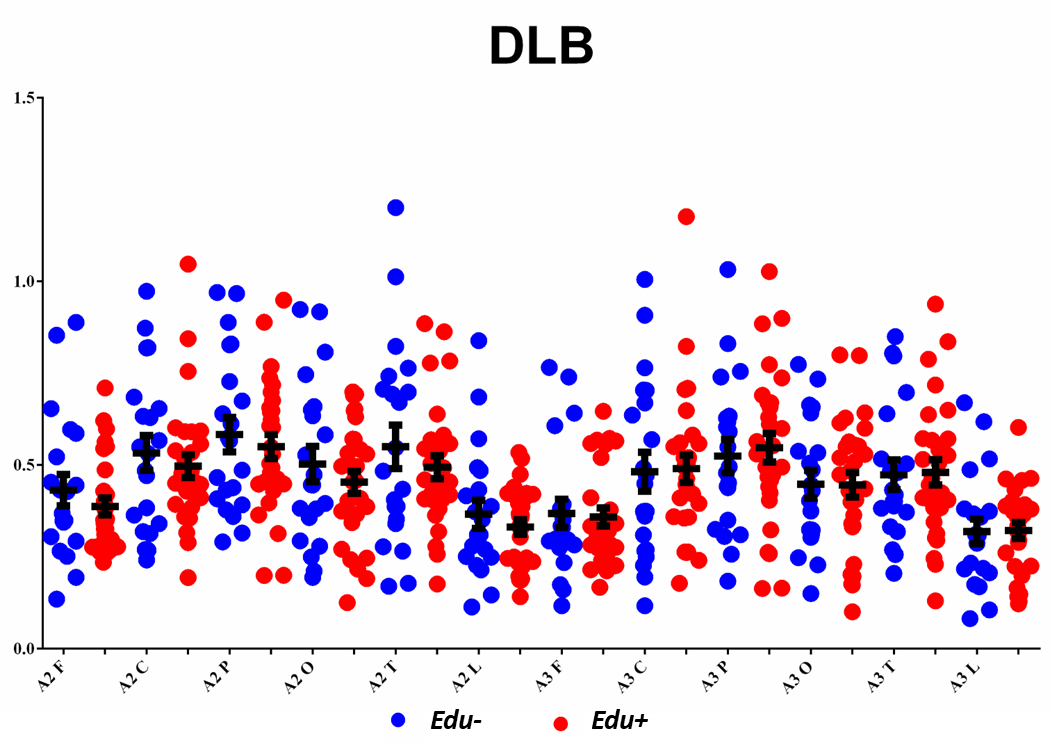


***Figure SM3.*** *Distribution of the individual values of the rsEEG alpha 2 and alpha 3 source activities (normalized eLORETA current density) in the DLB-Edu- and DLB-Edu+ subgroups. No outliers were detected according to Grubbs’ test with an arbitrary threshold of p > 0.001. Legend: DLB = Dementia due to Lewy Body disease; rsEEG = resting state electroencephalographic.*

## Topographic and frequency distribution of rsEEG source activities in the Edu- and Edu+ subgroups of Healthy, PDD, and DLB participants

For descriptive purposes, we reported the figures illustrating the topographic and frequency distribution of the rsEEG source activities for all the Healthy (Figure SM4) and DLB (Figure SM5) Edu- and Edu+ subgroups.

***Figure SM4***

***
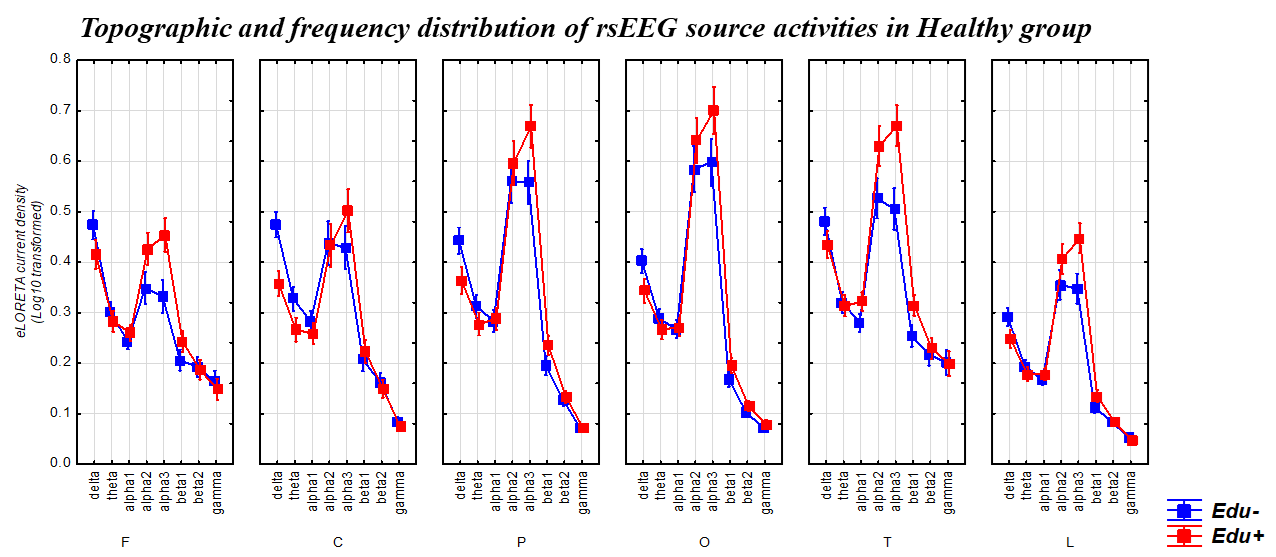
***

***Figure SM4.*** *Mean values (± standard error of the mean SE, log-10 transformed) of rsEEG source activities (normalized eLORETA current density) in Healthy participants according to the factors Education (Healthy-Edu- and Healthy-Edu+; dependent variable), Band (delta, theta, alpha 1, alpha 2, alpha 3, beta 1, beta 2, and gamma), and ROI (frontal, central, parietal, occipital, temporal, and limbic). The correspondent ANOVA was not statistically significant for the 3-way interaction among the factors Education, Band, and ROI ( p > 0.05). Legend: Healthy = cognitively unimpaired older persons; rsEEG = resting state electroencephalographic; ROI: Regions of Interest.*

***Figure SM5***


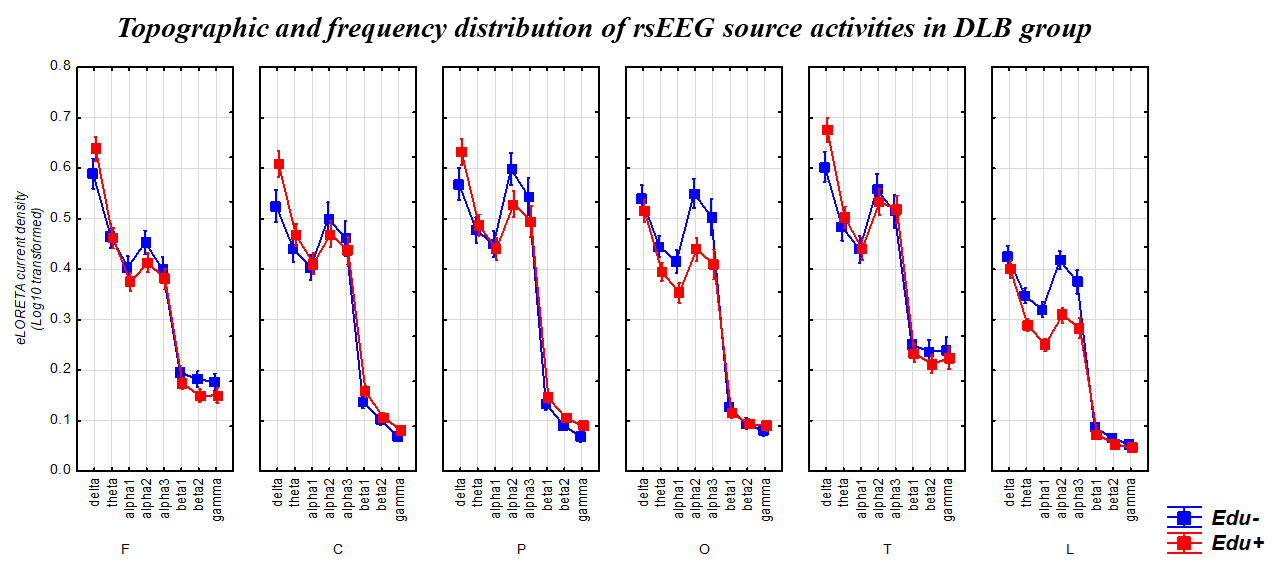


***Figure SM5.*** *Mean values (± standard error of the mean SE, log-10 transformed) of rsEEG source activities (normalized eLORETA current density) in DLB participants according to the factors Education (DLB-Edu- and DLB-Edu+; dependent variable), Band (delta, theta, alpha 1, alpha 2, alpha 3, beta 1, beta 2, and gamma), and ROI (frontal, central, parietal, occipital, temporal, and limbic). The correspondent ANOVA was not statistically significant for the 3-way interaction among the factors Education, Band, and ROI ( p > 0.05). Legend: DLB = Dementia due to Lewy Body disease; rsEEG = resting state electroencephalographic; ROI: Regions of Interest.*

*Control analysis on the effect of participants’ linguistic-cultural background*

As mentioned in the main article, a control analysis (p < 0.05) tested the effect of the participants’ linguistic-cultural background as a factor on the relationship between educational attainment and rsEEG rhythms in the Healthy, DLB, and PDD groups. For each group of participants (Healthy, DLB, and PDD), the control ANOVA (p < 0.05) included the factors of Linguistic-cultural background (Europe and Turkey), Education (Edu- and Edu+), Band (delta, theta, alpha 1, alpha 2, alpha 3, beta 1, beta 2, gamma), and ROI (frontal, central, parietal, occipital, temporal, and limbic). The rsEEG source activities (i.e., regionally normalized eLORETA solutions) were the dependent variable. Sex and global cognition (MMSE score) were used as covariates. The data were controlled for Gaussian distribution, as reported in the main article. Post-hoc comparisons were performed using the Duncan test (p < 0.05).

Figure SM6 illustrates the results of these analyses for the Healthy (left) and PDD (right) participants. No main or interaction effect that included the Linguistic-cultural background factor was observed (p > 0.05), indicating that the linguistic-cultural background did not affect the outcomes of the main statistical analyses in the present design.


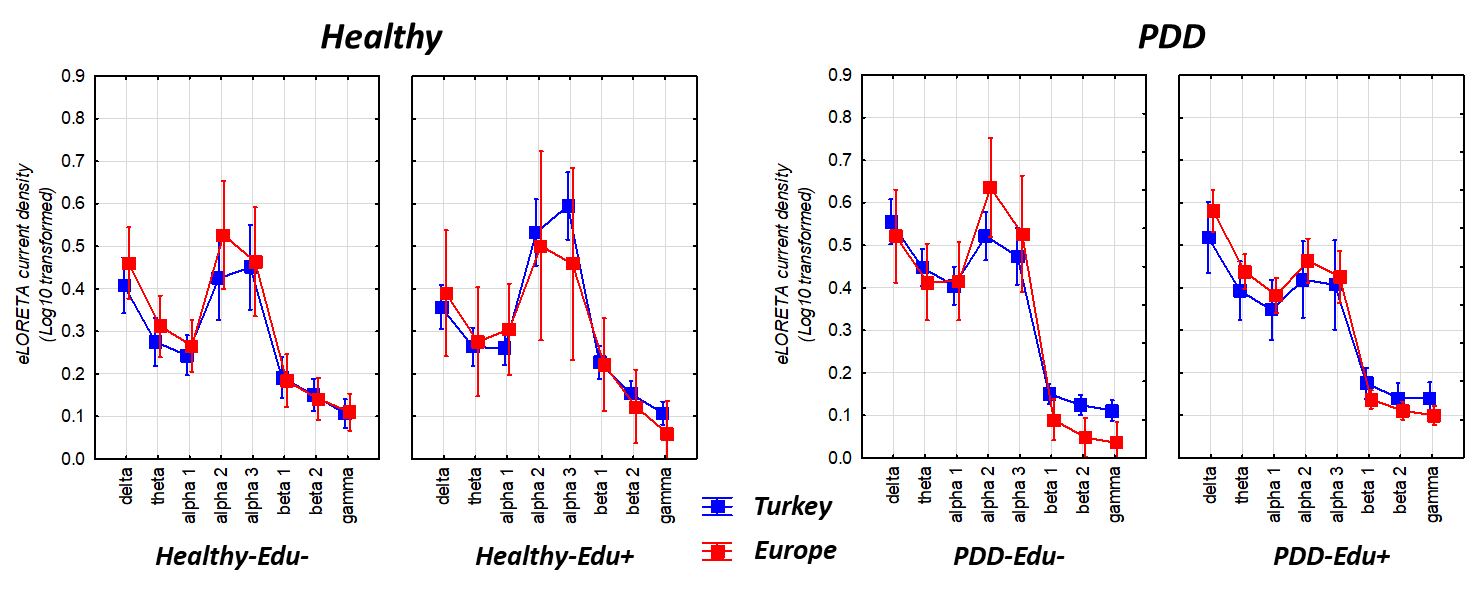


***Figure SM6.*** *Mean values (± standard error of the mean SE, log-10 transformed) of rsEEG source activities (normalized eLORETA current density) relative to not statistically significant ANOVA interaction effects in the Healthy (F = 7.45, p > 0.05, left) and PDD (F = 1.26, p > 0.05; right) between the factors of Education (Edu- and Edu+), Linguistic-cultural background (Europe, Turkey), and Band (delta, theta, alpha 1, alpha 2, alpha 3, beta 1, beta 2, and gamma). The sex and global cognition (MMSE) scores were used as covariates. Legend: Healthy = cognitively unimpaired older persons; PDD = Parkinson’s Disease Dementia; rsEEG = resting state electroencephalographic; MMSE = Mini-Mental State Evaluation.*

*Control analysis matching the educational attainment threshold defining the low and high-education sub-groups within the Healthy, DLB, and PDD participants*

As mentioned in the main article, a control analysis (p < 0.05) tested the effect of possible bias due to the different thresholds used to stratify the low (Edu-) and high (Edu+) education subgroups in the Healthy, DLB, and PDD groups of the main statistical analysis. For this purpose, this analysis matched the educational attainment levels of the Edu- and Edu+ subgroups across the Healthy, DLB, and PDD participants. Table SM1 summarizes the relevant demographic (i.e., age, education, and sex) and clinical (i.e., raw MMSE score and Unified Parkinson Disease Rating Scale-III; UPDRS III) score information about the Healthy-Edu- (N = 16), Healthy-Edu+ (N = 17), DLB-Edu- (N = 21), DLB-Edu+ (N = 19), PDD-Edu- (N = 16), PDD-Edu+ (N = 20) obtained by matching the educational attainment of the Edu- and Edu+ subgroups among the Healthy, DLB, and PDD groups. For each subgroup of participants (Healthy, DLB, and PDD), the control ANOVA (p < 0.05) included the factors of Education (Edu- and Edu+), Band (delta, theta, alpha 1, alpha 2, alpha 3, beta 1, beta 2, gamma), and ROI (frontal, central, parietal, occipital, temporal, and limbic). The rsEEG source activities (i.e., regionally normalized eLORETA solutions) were the dependent variable. Sex and global cognition (MMSE score) were used as covariates. The data were controlled for Gaussian distribution, as reported in the main article. No statistically significant differences among the Healthy, DLB, and PDD Edu- and Edu+ subgroups were observed (one-way ANOVA: education as the dependent variable, Group as a factor; p > 0.05) concerning the educational attainment measured in years.

***Table SM1***

| **DEMOGRAPHIC AND CLINICAL DATA** | | | |
| --- | --- | --- | --- |
|  | **Healthy-Edu-** | **Healthy-Edu+** | **Statistical analyses** |
| N | 16 | 17 | **-** |
| **Age (years)** | 64.2 ± 1.2 SE | 67.5 ± 1.8 SE | T test: n.s. |
| **Sex (M/F)** | 5/11 (31%) | 9/8 (53%) | Fisher test: n.s. |
| **Education (years)** | 5.3 ± 0.7 SE | 13.0 ± 0.4 SE | T test: p < 0.01 |
| **MMSE score** | 28.1 ± 0.5 SE | 28.5 ± 1.3 SE | Mann Whitney U test: p = n.s. |
| **MMSEc score** | 27.6 ± 0.4 SE | 27.3 ± 0.4 SE | Mann Whitney U test: p = n.s. |
|  |  |  |  |
|  | **DLB-Edu-** | **DLB-Edu+** | **Statistical analyses** |
| N | 21 | 19 | - |
| **Age (years)** | 74.0 ± 1.8 SE | 75.4 ± 2.0 SE | T test: p =n.s. |
| **Sex (M/F)** | 12/9 (41%) | 13/6 (68%) | Fisher test: n.s. |
| **Education (years)** | 5.0 ± 0.5 SE | 12.3 ± 0.4 SE | T test: p < 0.01 |
| **MMSE score** | 19.1 ± 1.3 SE | 21.3 ± 1.2 SE | Mann Whitney U test: p = n.s. |
| **MMSEc score** | 18.8 ± 1.3 SE | 19.8 ± 1.3 SE | Mann Whitney U test: p = n.s. |
| **UPDRS III score** | 13.5 ± 1.6 SE | 15.6 ± 2.0 SE | T test: n.s. |
|  |  |  |  |
|  | **PDD-Edu-** | **PDD-Edu+** | **Statistical analyses** |
| N | 16 | 20 | - |
| **Age (years)** | 71.8 ± 1.8 SE | 72.9 ± 1.1 SE | T test: p =n.s. |
| **Sex (M/F)** | 13/3 (81%) | 16/4 (83%) | Fisher test: n.s. |
| **Education (years)** | 4.3 ± 0.7 SE | 12.6 ± 0.2 SE | T test: p < 0.01 |
| **MMSE score** | 18.2 ± 1.0 SE | 22.2 ± 1.0 SE | Mann Whitney U test: p < 0.01 |
| **MMSEc score** | 18.1 ± 1.0 SE | 20.7 ± 1.0 SE | Mann Whitney U test: : p < 0.05 |
| **UPDRS III score** | 42.9 ± 0.6 SE | 39.6 ± 3.8 SE | T test: n.s. |

***Table SM1****. Mean values (± standard error of the mean, SE) of the demographic and clinical data as well as the results of their statistical comparisons (p < 0.05) in the Edu- and Edu+ subgroups of the Healthy, DLB, and PD participants matching the threshold for the stratification according to the low (Edu-) and high (Edu+) educational attainment subgroups. Legend: Healthy = cognitively unimpaired older persons; PDD = Parkinson’s Disease Dementia; DLB = Dementia due to Lewy Body disease; M/F = males/females; MMSE = Mini-Mental State Evaluation; MMSEc = Mini-Mental State Evaluation corrected for age and educational attainment; UPDRS III = Unified Parkinson Disease Rating Scale-III; n.s. = not significant (p > 0.05).*

The ANOVAs performed on this data subset confirmed the main results on the global cohort, illustrating that the discriminant pattern Healthy-Edu+ > Healthy-Edu- was fitted by the rsEEG alpha 3 (p < 0.05 Bonferroni corrected) source activities (Figure SM7 left). On the contrary, the discriminant pattern PDD-Edu- > PDD-Edu+ was fitted by the rsEEG alpha 2 and alpha 3 (p < 0.05 Bonferroni corrected) source activities (Figure SM7 right). No statistically significant effects were observed for the DLB group (p > 0.05).

**
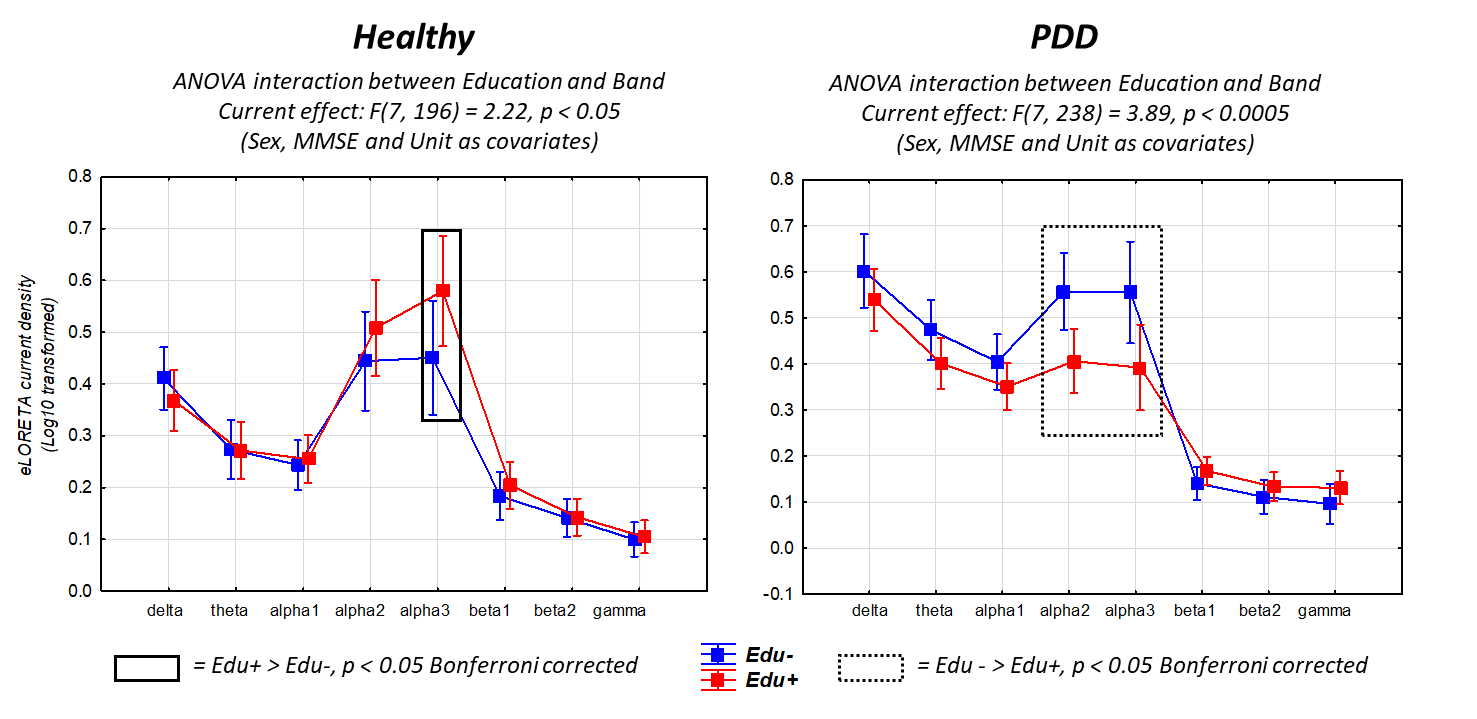
**

***Figure SM7.*** *Mean values (± standard error of the mean, SE, log-10 transformed) of rsEEG source activities (normalized eLORETA current density) relative to statistically significant ANOVA interaction effects in the Healthy (F = 2.22, p < 0.05, left) and PDD (F = 3.89, p < 0.01; right) between the factors Education (Edu- and Edu+) and Band (delta, theta, alpha 1, alpha 2, alpha 3, beta 1, beta 2, and gamma). The sex, MMSE, and clinical unit were used as a covariate. The rectangles indicate the frequency bands in which the eLORETA solutions statistically presented a significant difference between Healthy-Edu- and Healthy-Edu+ (p < 0.05 Bonferroni corrected; left) and between PDD-Edu- and PDD-Edu+ (p < 0.05 Bonferroni corrected; right). Legend: Healthy = cognitively unimpaired older persons; PDD = Parkinson’s Disease Dementia; rsEEG = resting state electroencephalographic.*

## Individual values of composite cognitive scores in Healthy, PDD, and DLB participants

The cognitive scores were calculated as z-scores in relation to the values of the Healthy group in the following cognitive domains: language, visuospatial, attention/executive, memory, and global (average among the previous ones) functions, as well as the raw MMSE. Figure SM7 reports the individual values of each Edu- and Edu+ subgroup of Healthy, PDD, and DLB participants. No outliers were identified according to Grubbs’ test (p > 0.001).

***Figure SM8***


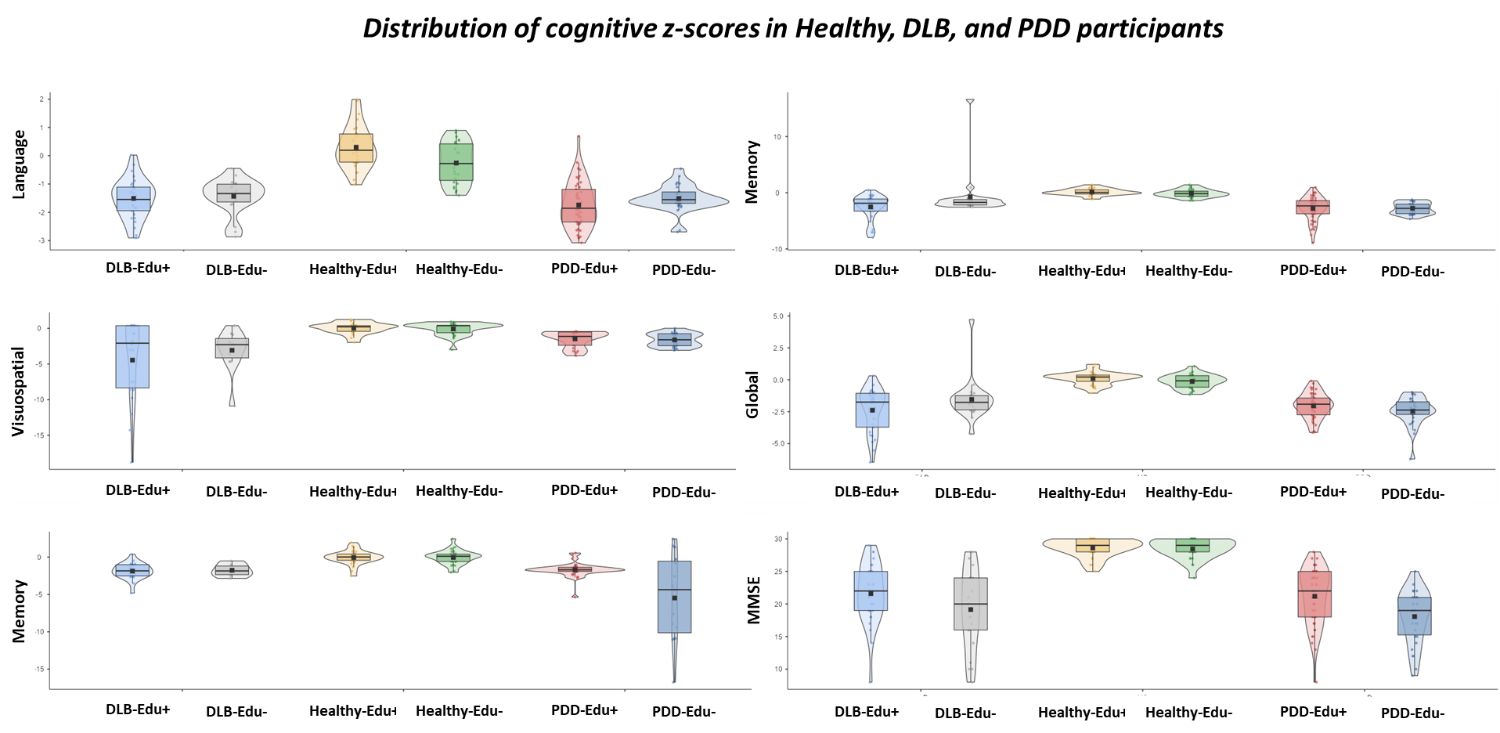


***Figure SM8.*** *Individual values of the composite cognitive scores calculated as z-scores in relation to the values of the Healthy group in the following cognitive domains: language, visuospatial, attention/executive, memory, and global (average among the previous ones) functions as well as the raw MMSE, in the Edu- and Edu+ subgroups of Healthy, PDD, and DLB participants. No outliers were identified according to Grubbs’ test (p > 0.001).Legend: MMSE = Mini-Mental State Evaluation.*

## Demographic, clinical, and rsEEG source markers in Healthy-Edu- and Healthy-Edu+ cross-validation subgroups

Table SM2 summarizes the relevant demographic (i.e., age, education, and sex) and clinical (i.e., MMSE score raw and corrected for age and education) information about the Healthy-Edu- (N = 27) and Healthy-Edu+ (N = 27) subgroups, together with the results of the statistical analyses computed to evaluate the presence or absence of statistically significant differences between these subgroups regarding age (T-test), sex (Fisher test), education (T-test), and MMSE score (raw and corrected; Mann-Whitney U-test). As expected, statistically significant differences were found between the Healthy-Edu- and Healthy-Edu+ subgroups for the education (p < 0.01). On the contrary, no statistically significant differences in age, sex, and MMSE score (raw and corrected) were found between the two subgroups (p > 0.05).

***Table SM2***

| **DEMOGRAPHIC AND CLINICAL DATA** | | | |
| --- | --- | --- | --- |
|  | **Healthy-Edu-** | **Healthy-Edu+** | **Statistical analyses** |
| **N** | 27 | 27 | **--** |
| **Age (years)** | 74.7 ± 1.0 SE | 73.3 ± 1.0 SE | T test: n.s. |
| **Sex (M/F)** | 12/15 (44%) | 12/15 (44%) | Fisher test: n.s. |
| **Education (years)** | 5.8 ± 0.4 SE | 13.2 ± 0.5 SE | T test: p < 0.01 |
| **MMSE score** | 28.5 ± 0.2 SE | 28.5 ± 0.2 SE | Mann Whitney U test: n.s. |
| **MMSEc score** | 27.8 ± 0.3 SE | 27.3 ± 0.3 SE | Mann Whitney U test: n.s. |

***Table SM2****. Mean values (± standard error of the mean, SE) of the demographic and clinical data as well as the results of their statistical comparisons (p < 0.05) in the subgroups of Healthy-Edu- (N = 27) and Healthy-Edu+ (N = 27) participants. Legend: Healthy = cognitively unimpaired older persons; M/F = males/females; MMSE = Mini-Mental State Evaluation; MMSEc = Mini-Mental State Evaluation corrected for age and educational attainment; n.s. = not significant (p > 0.05).*

The mean TF was 5.6 Hz (± 0.2 SE) in the Healthy-Edu- subgroup and 5.8 Hz (± 0.2 SE) in the Healthy-Edu+ subgroup. Furthermore, the mean IAF was 8.9 Hz (± 0.2 SE) in the Healthy-Edu- subgroup and 9.0 Hz (± 0.2 SE) in the Healthy-Edu+ subgroup. Two T-tests (p < 0.05) were performed to evaluate the presence or absence of statistically significant differences between the Healthy-Edu- and Healthy-Edu+ subgroups regarding TF and IAF. No statistically significant differences were found between the two subgroups (p > 0.05).

Figure SM8 shows the mean values (± SE, log-10 transformed) of rsEEG source activities (normalized eLORETA current density) relative to a statistically significant 2-way ANOVA interaction effect (F(7, 343) = 3.1610, p < 0.01) between the factors Education (Healthy-Edu- and Healthy-Edu+; dependent variable) and Band (delta, theta, alpha 1, alpha 2, alpha 3, beta 1, beta 2, and gamma). The Duncan planned post-hoc (p < 0.05 Bonferroni correction for 7 frequency bands, p < 0.05/7 = 0.007) testing showed that the discriminant pattern Healthy-Edu+> Healthy-Edu- was fitted by the rsEEG alpha 3 (p < 0.05 corrected) source activities. On the contrary, the rsEEG delta source activities were higher in the Healthy Edu- as compared to the Healthy-Edu+ subgroup (p < 0.05 corrected). No other effects involving the factor of Education were observed (p > 0.05).

***Figure SM9***

***
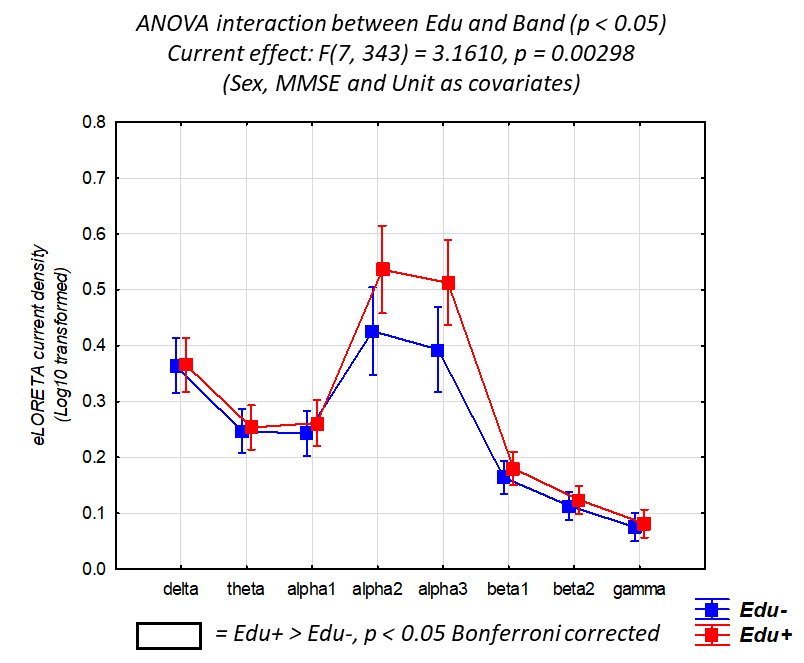
***

***Figure SM9.*** *Mean values (± standard error of the mean SE, log-10 transformed) of rsEEG source activities (normalized eLORETA current density) relative to a statistically significant ANOVA interaction effect (F = 3.16, p < 0.01) between the factors Education (Healthy-Edu-, N = 27; and Healthy-Edu+, N = 27) and Band (delta, theta, alpha 1, alpha 2, alpha 3, beta 1, beta 2, and gamma). The sex, MMSE, and clinical unit were used as a covariate. The rectangles indicate the frequency bands in which the eLORETA solutions statistically presented a significant difference between Healthy-Edu- and Healthy-Edu+ (p < 0.05 Bonferroni corrected). Legend: Healthy = cognitively unimpaired older persons; rsEEG = resting state electroencephalographic.*

The findings mentioned above were not due to outliers from individual normalized eLORETA current densities (log 10 transformed), as shown by Grubbs' test with an arbitrary threshold of p > 0.001.

## Demographic, clinical, and rsEEG source markers in PD(MCI+D)-Edu- and PD(MCI+D)-Edu+ subgroups

Table SM3 summarizes the relevant demographic (i.e., age, education, and sex) and clinical (i.e., MMSE score raw and corrected for age and education, visual hallucination, VH, and Unified Parkinson Disease Rating Scale-III, UPDRS III score; REM behavioral disorder, RBD) information about the PD(MCI+D)-Edu- (N = 19) and PD(MCI+D)-Edu+ (N = 19) subgroups, together with the results of the statistical analyses computed to evaluate the presence or absence of statistically significant differences between these subgroups regarding age (T-test), sex (Fisher test), education (T-test), MMSE score (raw and corrected; Mann-Whitney U-test), VH (Fisher test), UPDRS III score (T-test). As expected, statistically significant differences were found between the PD(MCI+D)-Edu- and PD(MCI+D)-Edu+ subgroups for the education (p < 0.01). MMSE score was higher in PD(MCI+D)-Edu+ than PD(MCI+D)-Edu- subgroups (p < 0.01). On the contrary, no statistically significant differences in age, sex, MMSE score corrected, VH, UPDRS III score, and RBD were found between the two subgroups (p > 0.05).

***Table SM3***

| **DEMOGRAPHIC AND CLINICAL DATA** | | | |
| --- | --- | --- | --- |
|  | **PD(MCI+D)-Edu-** | **PD(MCI+D)-Edu+** | **Statistical analyses** |
| N | 19 | 19 | - |
| **Age (years)** | 70.6 ± 1.6 SE | 68.1 ± 1.3 SE | T-test: n.s. |
| **Sex (M/F)** | 13/6 (68%) | 15/4 (79%) | Fisher test: n.s. |
| **Education (years)** | 4.8 ± 0.5 SE | 13.2 ± 0.5 SE | T-test: p < 0.0005 |
| **MMSE score** | 20.9 ± 0.7 SE | 21.9 ± 1.4 SE | Mann Whitney U test:  p < 0.01 |
| **MMSEc score** | 21.3 ± 0.9 SE | 23.6 ± 1.3 SE | Mann Whitney U test: n.s. |
| **VH (%)** | 42% | 32% | Fisher test: n.s. |
| **UPDRS III** | 28.1 ± 3.4 SE | 30.6 ± 3.8 SE | T-test: n.s. |
| **RBD (%)** | 53% | 51% | Fisher test: n.s. |

***Table SM3****. Mean values (± SE) of the demographic and clinical data as well as the results of their statistical comparisons (p < 0.05) in the subgroups of PD(MCI+D)-Edu- (N = 19) and PD(MCI+D)-Edu+ (N = 19) participants. Legend: PD(MCI+D) = Parkinson’s Disease with Mild Cognitive Impairment or Dementia; M/F = males/females; MMSE = Mini-Mental State Evaluation; MMSEc = Mini-Mental State Evaluation corrected for age and educational attainment; VH = visual hallucination; UPDRS III = Unified Parkinson Disease Rating Scale-III; RBD = REM behavioral disorder; n.s. = not significant (p > 0.05).*

The mean TF was 4.5 Hz (± 0.1 SE) in the PDD-Edu- subgroup and 4.7 Hz (± 0.1 SE) in the PD(MCI+D)-Edu+ subgroup. Furthermore, the mean IAF was 7.2 Hz (± 0.2 SE) in the PD(MCI+D)-Edu- subgroup and 6.7 Hz (± 0.1 SE) in the PDD-Edu+ subgroup. Two T-tests (p < 0.05) were performed to evaluate the presence or absence of statistically significant differences between the PD(MCI+D)-Edu- and PD(MCI+D)-Edu+ subgroups regarding TF and IAF. No statistically significant differences were found between the two subgroups (p > 0.05).

Figure SM9 shows the mean values (± SE, log-10 transformed) of rsEEG source activities (normalized eLORETA current density) relative to a statistically significant 2-way ANOVA interaction effect (F(7, 231) = 2.1567, p < 0.05) between the factors Education (PD(MCI+D)-Edu- and PD(MCI+D)-Edu+; dependent variable) and Band (delta, theta, alpha 1, alpha 2, alpha 3, beta 1, beta 2, and gamma). The Duncan planned post-hoc (p < 0.05 Bonferroni correction for 7 frequency bands, p < 0.05/7 = 0.007) testing showed that the discriminant pattern PD(MCI+D)-Edu- > PD(MCI+D)-Edu+ was fitted by the rsEEG alpha 3 (p < 0.05, Bonferroni corrected) source activity. No other effects involving the factor of Education were observed (p > 0.05).

***Figure SM10***

***
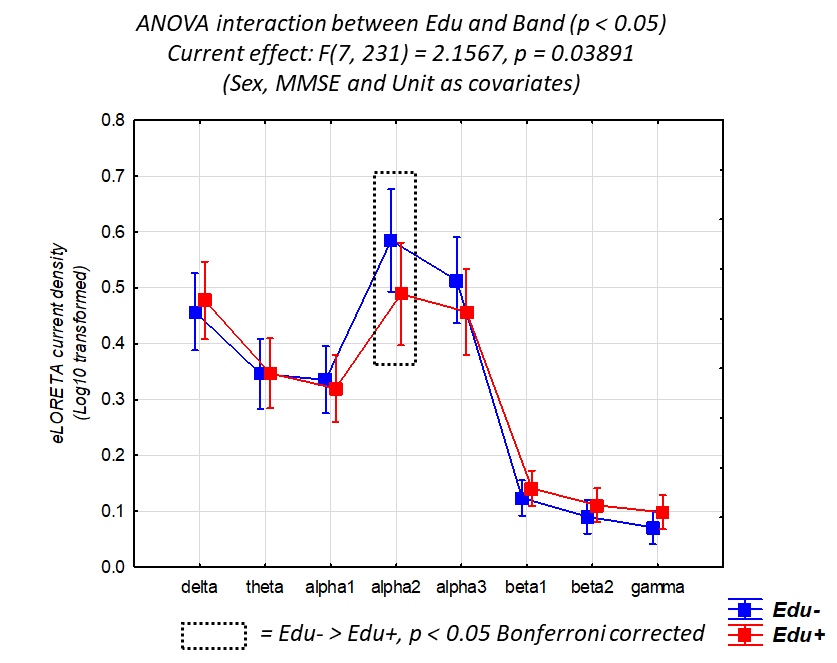
***

***Figure SM10.*** *Mean values (±SE, log-10 transformed)* *of rsEEG source activities (normalized eLORETA current density) relative to a statistically significant ANOVA interaction effect (F =2.16, p < 0.05) between the factors Education (PD(MCI+D)-Edu-, N = 19; and PD(MCI+D)-Edu+, N = 19) and Band (delta, theta, alpha 1, alpha 2, alpha 3, beta 1, beta 2, and gamma). The sex, MMSE, and clinical unit were used as a covariate. The rectangles indicate the frequency bands in which the eLORETA solutions statistically presented a significant difference between PD(MCI+D)-Edu- and PD(MCI+D)-Edu+ (p < 0.05 Bonferroni corrected). Legend: PD(MCI+D) = Parkinson’s Disease Mild Cognitive Impairment or Dementia; rsEEG = resting state electroencephalographic.*

The findings mentioned above were not due to outliers from individual normalized eLORETA current densities (log 10 transformed), as shown by Grubbs' test with an arbitrary threshold of p > 0.001.

## Demographic, clinical, and rsEEG source markers in DLB(MCI+D)-Edu- and DLB(MCI+D)-Edu+ subgroups

Table SM4 summarizes the relevant demographic (i.e., age, education, and sex) and clinical (i.e., MMSE score raw and corrected for age and education, visual hallucination, VH, and Unified Parkinson Disease Rating Scale-III, UPDRS III score; REM behavioral disorder, RBD) information about the DLB(MCI+D)-Edu- (N = 19) and DLB(MCI+D)-Edu+ (N = 19) subgroups, age (T-test), sex (Fisher test), education (T-test), MMSE score (raw and corrected; Mann-Whitney U-test), VH (Fisher test), UPDRS III score (T-test). As expected, statistically significant differences were found between the DLB(MCI+D)-Edu- and DLB(MCI+D)-Edu+ subgroups for education (p < 0.01). On the contrary, no statistically significant differences in age, sex, MMSE score (raw and corrected), VH, UPDRS III score, and RBD were found between the two subgroups (p > 0.05).

***Table SM4***

| **DEMOGRAPHIC AND CLINICAL DATA** | | | |
| --- | --- | --- | --- |
|  | **DLB(MCI+D)-Edu-** | **DLB(MCI+D)-Edu+** | **Statistical analyses** |
| **N** | 22 | 22 | - |
| **Age (years)** | 76.0 ± 0.9 SE | 75.8 ± 1.4 SE | T test: n.s. |
| **Sex (M/F)** | 12/10 (55%) | 15/7 (68%) | Fisher test: n.s. |
| **Education (years)** | 5.1 ± 0.5 SE | 12.3 ± 0.6 SE | T test: p < 0.01 |
| **MMSE score** | 21.0 ± 1.0 SE | 21.4 ± 0.6 SE | Mann Whitney U test: n.s. |
| **MMSEc score** | 21.1 ± 1.0 SE | 22.5 ± 0.7 SE | Mann Whitney U test: n.s. |
| **VH (%)** | 64% | 62% | Fisher test: n.s. |
| **UPDRS III** | 21.9 ± 3.1 SE | 19.0 ± 2.3 SE | T test: n.s. |
| **RBD (%)** | 77% | 55% | Fisher test: n.s. |

***Table SM4****. Mean values (± SE) of the demographic and clinical data as well as the results of their statistical comparisons (p < 0.05) in the subgroups of DLB(MCI+D)-Edu- (N = 22) and DLB(MCI+D)-Edu+ (N = 22) participants. Legend: DLB(MCI+D) = Mild Cognitive Impairment or Dementia due to Lewy Body disease; M/F = males/females; MMSE = Mini-Mental State Evaluation; MMSEc = Mini-Mental State Evaluation corrected for age and educational attainment; VH = visual hallucination; UPDRS III = Unified Parkinson Disease Rating Scale-III; RBD = REM behavioral disorder; n.s. = not significant (p > 0.05).*

The mean TF was 4.7 Hz (± 0.2 SE) in the DLB-Edu- subgroup and 4.8 Hz (± 0.2 SE) in the DLB-Edu+ subgroup. Furthermore, the mean IAF was 7.6 Hz (± 0.3 SE) in the DLB-Edu- subgroup and 7.0 Hz (± 0.2 SE) in the DLB-Edu+ subgroup. Two T-tests (p < 0.05) were performed to evaluate the presence or absence of statistically significant differences between the DLB-Edu- and DLB-Edu+ subgroups regarding TF and IAF. No statistically significant differences were found between the two subgroups (p > 0.05).

The ANOVA did not show any statistically significant 3- or 2-way interaction effect (p > 0.05) between the factors Education (DLB(MCI+D)-Edu- and DLB(MCI+D)-Edu+; dependent variable), Band (delta, theta, alpha 1, alpha 2, alpha 3, beta 1, beta 2, and gamma), and ROI (frontal, central, parietal, occipital, temporal, and limbic).

The findings mentioned above were not due to outliers from individual normalized eLORETA current densities (log 10 transformed), as shown by Grubbs' test with an arbitrary threshold of p > 0.001.

## Correlation between motor impairment and rsEEG source activities

To evaluate whether the compensatory mechanism revealed by rsEEG rhythms in PD(MCI+D) group may depend on motor dysfunction, we explored the correlation between the motor impairment (UPDRS III) and the theta and alpha rsEEG source activities. The theta band was included as associated with greater motor impairment in PD patients when recorded from sensorimotor cortical areas (Karimi et al., 2021).

To this aim, we developed several Pearson’s correlation models between the UPDRS III score and the regional (frontal, central, parietal, occipital, and temporal) and global rsEEG theta, alpha 2, and alpha 3 source activities in PD(MCI+D) and DLB(MCI+D) groups. To include as many patients as possible, we pulled together the experimental and the cross-validation cohorts. Only for the PD(MCI+D) group (N = 104) statistically significant positive correlations were observed between the regional and global rsEEG theta source activities and the UPDRS III score with a Pearson’s r ranging between 0.19-0.30 (p < 0.05; Figure SM10).

***Figure SM11***

***
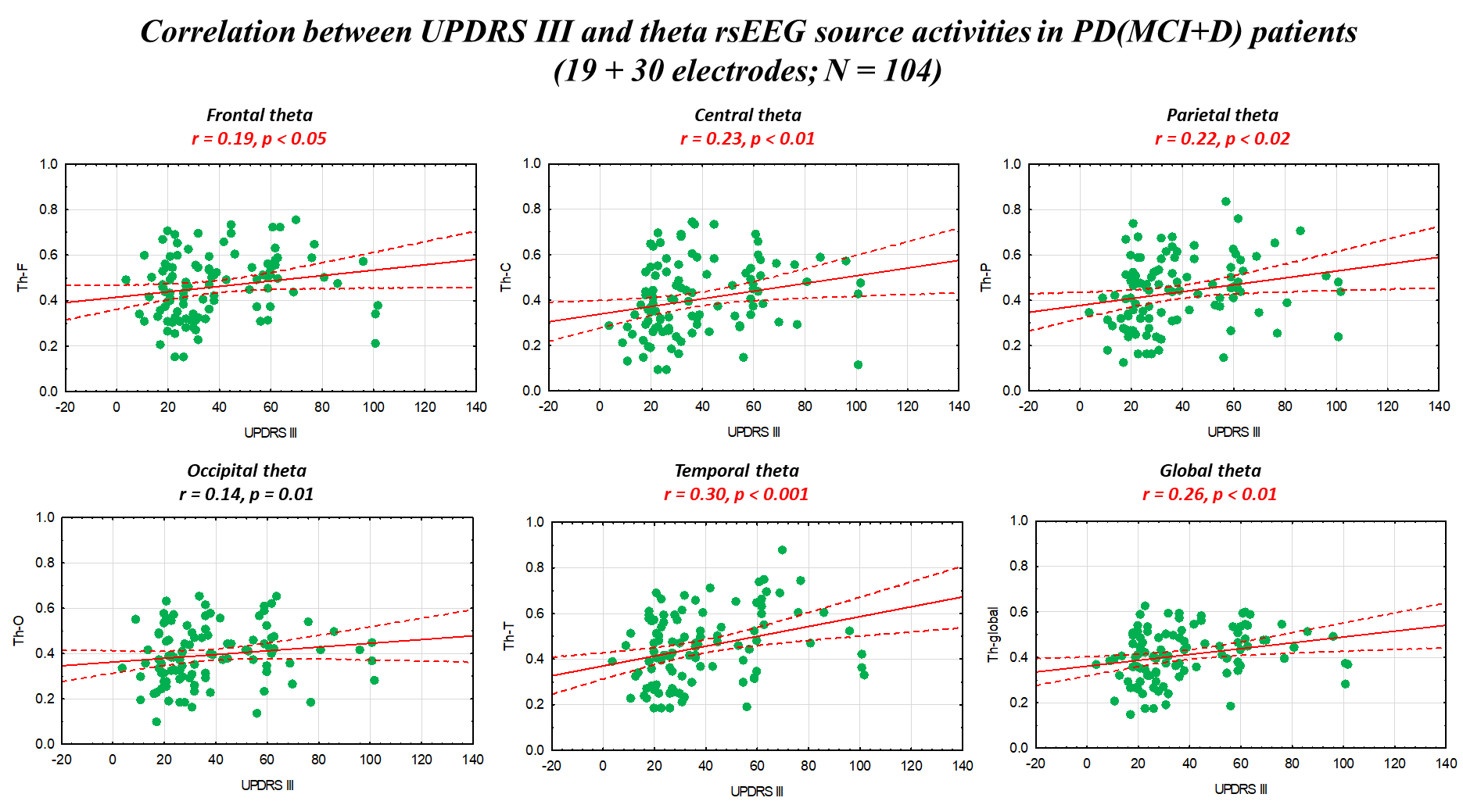
***

***Figure SM11.*** *Plots of the correlation analysis between theta rsEEG source activities and motor impairment as revealed by UPDRS III in PD(MCI+D) participants. Statistically significant effects are highlighted in red. Legend: PD(MCI+D) = Parkinson’s Disease Mild Cognitive Impairment or Dementia; UPDRS III = Unified Parkinson Disease Rating Scale-II).*

No statistically significant correlations were observed for the DLB group (N = 83; p > 0.05; Figure SM11).

***Figure SM12***

***
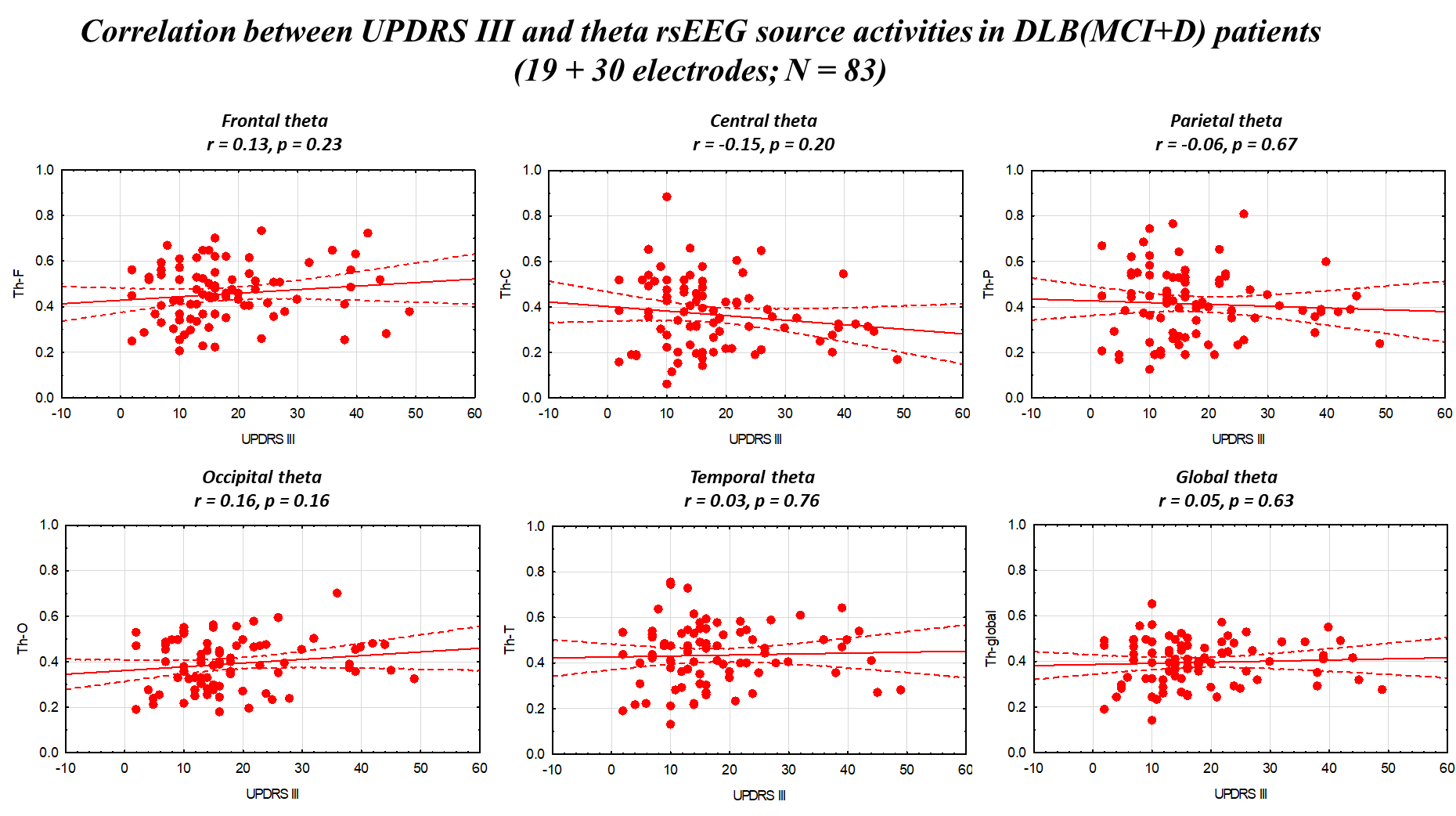
***

***Figure SM12.*** *Plots of the correlation analysis between theta rsEEG source activities and motor impairment as revealed by UPDRS III in DLB(MCI+D) participants. Statistically significant effects are highlighted in red. Legend: DLB(MCI+D) = Mild Cognitive Impairment or Dementia due to Lewy Body disease; UPDRS III = Unified Parkinson Disease Rating Scale-III.*

Concerning rsEEG alpha 2 and alpha 3 source activities, no statistically significant correlations were observed for PD(MCI+D) and DLB(MCI+D), as reported in Table SM5 and Table SM6, respectively.

***Table SM5***

| **CORRELATIONS BETWEEN rsEEG ALPHA AND UPDRS III IN PD(MCI+D)** | | |
| --- | --- | --- |
|  | **rsEEG Alpha 2** | **rsEEG Alpha 3** |
| **F** | r = 0.15, p = n.s. | r = 0.10, p = n.s. |
| **C** | r = -0.01, p = n.s. | r = -0.06, p = n.s. |
| **P** | r = -0.07, p = n.s. | r = -0.12, p = n.s. |
| **O** | r = -0.11, p = n.s. | r = -0.10, p = n.s. |
| **T** | r = 0.11, p = n.s. | r = 0.08, p = n.s. |
| **Global** | r = -0.02, p = n.s. | r = -0.02, p = n.s. |

***Table SM5.*** *Results of the correlation analysis between alpha 2 and rsEEG alpha 3 source activities and motor impairment as revealed by UPDRS III in PD(MCI+D) participants. The main and cross-validation cohorts were pulled together. The Pearson’s r and the relative p-value are reported for each pair of variables. Legend: PD(MCI+D) = Parkinson’s Disease with Mild Cognitive Impairment or Dementia; UPDRS III = Unified Parkinson Disease Rating Scale-III; n.s. = not significant (p > 0.05).*

***Table SM6***

| **CORRELATIONS BETWEEN rsEEG ALPHA AND UPDRS III IN DLB(MCI+D)** | | |
| --- | --- | --- |
|  | **rsEEG Alpha 2** | **rsEEG Alpha 3** |
| **F** | r = 0.02, p = n.s. | r = 0.05, p = n.s. |
| **C** | r = -0.12, p = n.s. | r = -0.13, p = n.s. |
| **P** | r = 0.05, p = n.s. | r = -0.03, p = n.s. |
| **O** | r = 0.22, p = n.s. | r = 0.16, p = n.s. |
| **T** | r = 0.07, p = n.s. | r = 0.04, p = n.s. |
| **Global** | r = 0.08, p = n.s. | r = 0.03, p = n.s. |

***Table SM6.*** *Results of the correlation analysis between rsEEG alpha 2 and alpha 3 source activities and motor impairment as revealed by UPDRS III in PD(MCI+D) participants. The main and cross-validation cohorts were pulled together. The Pearson’s r and the relative p-value are reported for each pair of variables. Legend: DLB(MCI+D) = Mild Cognitive Impairment or Dementia due to Lewy Body disease; UPDRS III = Unified Parkinson Disease Rating Scale-III; n.s. = not significant (p > 0.05).*

**References**

1. Gelb DJ, Oliver E, Gilman S. Diagnostic criteria for Parkinson disease. Arch Neurol. 1999;56(1):33-9. <https://doi.org/10.1001/archneur.56.1.33>
2. Hoehn MM, Yahr MD. Parkinsonism: onset, progression and mortality. Neurology. 1967;17(5):427-42. <https://doi.org/10.1212/wnl.17.5.427>
3. Fahn S, Elton RL, Members of the UPDRS Development Committee. The Unified Parkinson’s Disease Rating Scale. In: Fahn S, Marsden CD, Calne DB, Goldstein M, editors. Recent developments in Parkinson’s disease. Vol. 2. McMellam Health Care Information; 1987. p. 153-63.
4. Litvan I, Aarsland D, Adler CH, Goldman JG, Kulisevsky J, Mollenhauer B, Rodriguez-Oroz MC, Tröster AI, Weintraub D. MDS Task Force on mild cognitive impairment in Parkinson's disease: critical review of PD-MCI. Mov Disord. 2011;26(10):1814-24. <https://doi.org/10.1002/mds.23823>
5. Geser F, Wenning GK, Poewe W, McKeith I. How to diagnose dementia with Lewy bodies: state of the art. Mov Disord. 2005;20 Suppl 12:S11-20. <https://doi.org/10.1002/mds.20535>
6. McKeith IG, Dickson DW, Lowe J, Emre M, O'Brien JT, Feldman H, Cummings J, Duda JE, Lippa C, Perry EK, Aarsland D, Arai H, Ballard CG, Boeve B, Burn DJ, Costa D, Del Ser T, Dubois B, Galasko D, Gauthier S, et al. Diagnosis and management of dementia with Lewy bodies: third report of the DLB Consortium. Neurology. 2005;65(12):1863-72. https://doi.org/10.1212/01.wnl.0000187889.17253.b1
7. McKeith IG, Galasko D, Kosaka K, Perry EK, Dickson DW, Hansen LA, Salmon DP, Lowe J, Mirra SS, Byrne EJ, Lennox G, Quinn NP, Edwardson JA, Ince PG, Bergeron C, Burns A, Miller BL, Lovestone S, Collerton D, Jansen EN, et al. Consensus guidelines for the clinical and pathologic diagnosis of dementia with Lewy bodies (DLB): report of the consortium on DLB international workshop. Neurology. 1996;47(5):1113-24. <https://doi.org/10.1212/wnl.47.5.1113>
8. Babiloni C, Del Percio C, Lizio R, Noce G, Cordone S, Lopez S, et al. Abnormalities of cortical neural synchronization mechanisms in patients with dementia due to Alzheimer's and Lewy body diseases: an EEG study. Neurobiol Aging. 2017;55:143-58.
9. McKeith IG, Boeve BF, Dickson DW, Halliday G, Taylor JP, Weintraub D, Aarsland D, Galvin J, Attems J, Ballard CG, Bayston A, Beach TG, Blanc F, Bohnen NI, Bonanni L, Bras J, Brundin P, Burn DJ, Chen-Plotkin A, Duda JE, et al. Diagnosis and management of dementia with Lewy bodies: Fourth consensus report of the DLB Consortium. Neurology. 2017;89(1):88-100. <https://doi.org/10.1212/WNL.0000000000004058>
10. Cummings JL, Mega M, Gray K, Rosenberg-Thompson S, Carusi DA, Gornbein J. The Neuropsychiatric Inventory: comprehensive assessment of psychopathology in dementia. Neurology. 1994;44(12):2308-14. <https://doi.org/10.1212/wnl.44.12.2308>
11. Dubois B, Slachevsky A, Litvan I, Pillon B. The FAB: a Frontal Assessment Battery at bedside. Neurology. 2000;55(11):1621-6. <https://doi.org/10.1212/wnl.55.11.1621>
12. Walker MP, Ayre GA, Cummings JL, Wesnes KA, McKeith IG, O'Brien JT, Ballard CG. The Clinician Assessment of Fluctuation and the One Day Fluctuation Assessment Scale. Two methods to assess fluctuating confusion in dementia. Br J Psychiatry. 2000;177:252-6.
13. Walker MP, Ayre GA, Perry EK, Wesnes KA, McKeith IG, Tovee MJ, Edwardson JA, Ballard CG. Quantification and characterization of fluctuating cognition in dementia with Lewy bodies and Alzheimer's disease. Dement Geriatr Cogn Disord. 2000;11(6):327-35.
14. Jurica PJ, Leitten CL, Mattis S. Dementia Rating Scale-2 (DRS-2). Psychological Assessment Resources; 2001.
15. Donaghy PC, Barnett N, Olsen K, Taylor JP, McKeith IG, O'Brien JT, Thomas AJ. Symptoms associated with Lewy body disease in mild cognitive impairment. Int J Geriatr Psychiatry. 2017;32(11):1163-71. <https://doi.org/10.1002/gps.4742>
16. Novelli G, Papagno C, Capitani E, Laiacona M, et al. Tre test clinici di ricerca e produzione lessicale. Taratura su soggetti normali [Three clinical tests to research and rate the lexical performance of normal subjects]. Arch Psicol Neurol Psichiatr. 1986;47(4):477-506.
17. Benton AL, Sivan AB, Hamsher KD, Varney NR, Spreen O. Facial recognition: Stimulus and multiple-choice pictures. In: Benton AL, Sivan AB, Hamsher KD, Varney NR, Spreen O, editors. Contributions to neuropsychological assessment. Oxford University Press; 1983. p. 30–40.
18. Reitan RM. Validity of the Trail Making Test as an Indicator of Organic Brain Damage. Percept Mot Skills. 1958;8(3):271-6. <https://doi.org/10.2466/pms.1958.8.3.271>
19. Stroop JR. Studies of interference in serial verbal reactions. J Exp Psychol. 1935;18(6):643-62. <https://doi.org/10.1037/h0054651>
20. Inouye SK, van Dyck CH, Alessi CA, Balkin S, Siegal AP, Horwitz RI. Clarifying confusion: the confusion assessment method. A new method for detection of delirium. Ann Intern Med. 1990;113(12):941-8. <https://doi.org/10.7326/0003-4819-113-12-941>
21. Wechsler D. WMS-R: Wechsler Memory Scale-Revised: Manual. San Antonio, TX: Psychological Corporation; 1987.
22. Öktem Ö. Sözel bellek süreçleri testi (bir ön çalışma). Nöropsikiyatri Arşivi. 1992;29:196-206.
